# Effect of Conformational Variability on Seasonable Thermal Stability and Cell Entry of Omicron Variants

**DOI:** 10.1021/acsomega.2c08075

**Published:** 2023-02-10

**Authors:** Hiroshi Izumi, Hiroshi Aoki, Laurence A. Nafie, Rina K. Dukor

**Affiliations:** † National Institute of Advanced Industrial Science and Technology (AIST), AIST Tsukuba West, Tsukuba, Ibaraki 305-8569, Japan; ‡ Department of Chemistry, Syracuse University, Syracuse, New York 13244-4100, United States; § BioTools, Inc., Bee Line Hwy, Jupiter, Florida 33458, United States

## Abstract

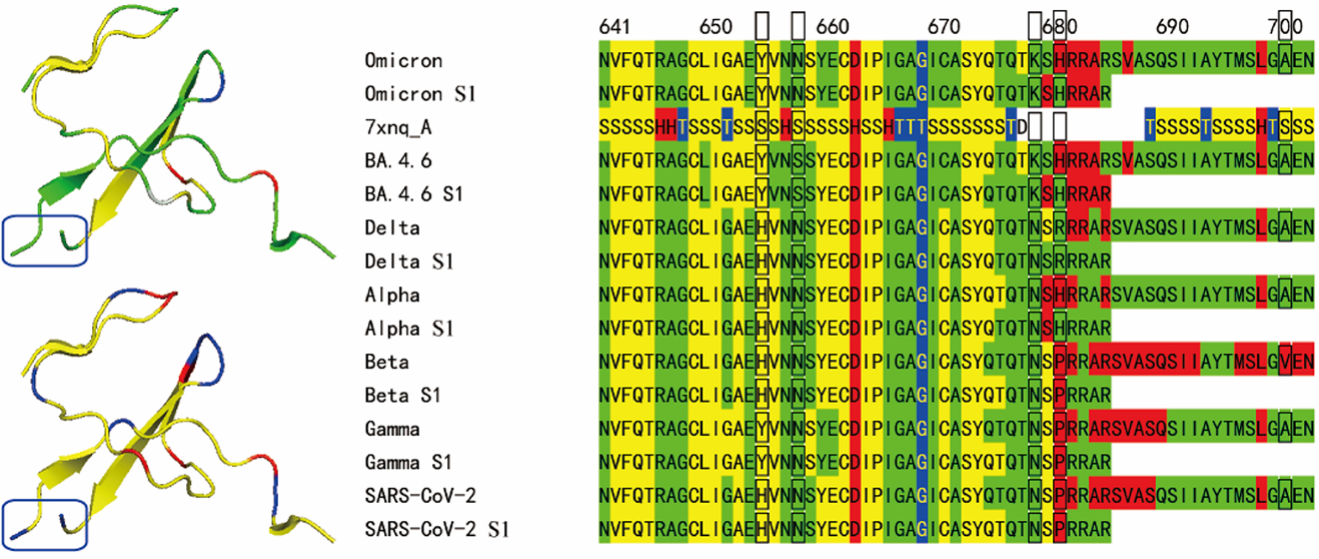

The Omicron BA.1 variant of SARS-CoV-2 preferentially infects through the cathepsin-mediated endocytic pathway, but the mechanism of cell entry has not been solved yet because BA.4/5 is more fusogenic and more efficiently spread in human lung cells than BA.2. It has been unclear why the Omicron spike is inefficiently cleaved in virions compared with Delta, and how the relatively effective reproduction proceeds without the cell entry through plasma membrane fusion. Conformational variability from deep neural network-based prediction correlates well with the thermodynamic stability of variants. The difference of seasonable pandemic variants in summer and those in winter is distinguishable by this conformational stability, and the geographical optimization of variants is also traceable. Further, the predicted conformational variability maps rationalize the less efficient S1/S2 cleavage of Omicron variants and provide a valuable insight into the cell entry through the endocytic pathway. It is concluded that conformational variability prediction is able to complement transformation information on motifs in protein structures for drug discovery.

## Introduction

Deep learning methods, such as Alphafold,^1^ Rosettafold,^2^ and ProteinMPNN,^3^ have predicted reliable protein structures and have had a great transformative effect on molecular biology. Recently, over 200 million entries of the predicted protein structures have been released on the AlphaFold database,^4^ but the spike protein structure of SARS-CoV-2 has not been found as far as we can see because of the flexibility of conformations. Actually, structural modeling of eight synthetic receptor binding domain (RBD) variants from deep mutational learning by AlphaFold2 has demonstrated that the angiotensin-converting enzyme 2-binding (ACE2-binding) variants show a wide diversity of possible structural conformations.^5^ We have reported the deep neural network-based conformational variability prediction system of protein structures (SSSCPreds) and the high correlation between conformational variability of RBD for SARS-CoV-2 spike protein and actual expression or ACE2 binding affinity.^6^ The analysis of D614G mutation with the predicted conformational variability has demonstrated that the left-handed α-helix-type conformation of G614 contributes to the reduction of S1 shedding, high expression, and increase in infectivity because glycine lacks chirality.^7^ The predicted conformational variability of RBD for Alpha, Beta, Gamma, and Delta variants against that of wild type (WT) also corresponds well to the relative feature of antibody evasion and expression for each variant.^7^

SSSCPreds can simultaneously predict locations of protein flexibility or rigidity and the shapes of those regions with high accuracy.^6^ The supersecondary structure code (SSSC) is represented as a conformation term using the letters “H”, “S”, “T”, and “D” for each amino acid peptide unit referring to an α-helix-type conformation (H), a β-sheet-type conformation (S), a variety of other-type conformations (T), and disordered residues or the C-terminus (D).^6^ This code has been approved as a protocol for molecular biology database and can be used to describe the characteristic loop structures, such as IgG immunoglobulin (SSSC: SHHSHSS) versus IgM rheumatoid factor (SSSC: TTTSSSS).^6^ The conformational variability is obtained from SSSCPreds that uses input from three structure prediction systems SSSCPred, SSSCPred100, and SSSCPred200, in which each system has the high average concordance rate (>0.86) of benchmarks using agreements of H, S, T, D symbols, respectively.^6^ The advantage of this method is that the modification of parameters for each system is not necessary so that only the input of protein sequences is needed. However, the prediction accuracy of conformational variability is low for proteins such as an influenza A (H3N2) hemeagglutinin at pH 5 with very few measurement conditions. At least, 100,000 subunit data at pH 5 are necessary to get a “good” prediction accuracy with a high concordance rate. However, users do not need to consider the measurement conditions except those for proteins placed in special environments such as antifreeze proteins.

The cryo-electron microscopy (cryo-EM) structures of Omicron variants have revealed the increased antibody evasion.^8−11^ In comparison with the similar replication for the Omicron BA.1 and Delta variants in human nasal epithelial cultures, the BA.1 variant has demonstrated lower replication in lung cells and gut cells.^12^ The difference is rationalized by the less efficient S1/S2 cleavage and inability to use transmembrane protease, serine 2 (TMPRSS2), and accordingly, it suggests greater dependency on cell entry for the BA.1 variant through the cathepsin-mediated endocytic pathway.^12,13^ However, although the Delta variant has the greatest fusogenicity and pathogenicity of the five SARSCoV-2 variants tested, BA.4/5 is more fusogenic and more efficiently spread in human lung cells than BA.2.^14^ The mechanism of cell entry has not been solved yet. It has not been explained in detail why the Omicron spike is inefficiently cleaved in virions compared with Delta^12^ and how approximately 3-fold enhanced binding affinity of Omicron BA.1 variant for ACE2^12^ is directly associated with the relative effective reproduction numbers three to five of BA.1 variant (relative to Delta) without the cell entry through plasma membrane fusion.^15^

The Omicron BA.2.75 variant emerged in India has K147E, W152R, F157L, I210V, G257S, G339H, G446S, N460K, and R493Q (reversion) mutations against BA.2^16^ and has been detected in at least 15 countries as of 19 July 2022.^17^ BA.2.75 shows moderately greater immune evasion than BA.2 but indicates a less pronounced degree of antibody evasion compared with BA.4/5.^16−18^ Alternatively, once the proportion of BA.4.6 had rose in Iowa, Kansas, Missouri, and Nebraska, then those of BQ.1.1, BQ. 1, BF.7, and BA.2.75.2 have gradually increased in United States.^19^

The K417N mutation of Beta variant circulated from South Africa with the more flexible SSSC sequence can escape neutralization more effectively than the K417T mutation of Gamma variant emerged in Brazil with the more rigid one.^7^ To define the effect of conformational variability on the Omicron variants in view of thermal stability, we analyzed the sequence flexibility/rigidity map using SSSCPreds with the reported cryo-EM and X-ray crystallographic structures of Omicron variants.

## Results and Discussion

### Furin Cleavage Site of Spike Protein

**Figure 1:**
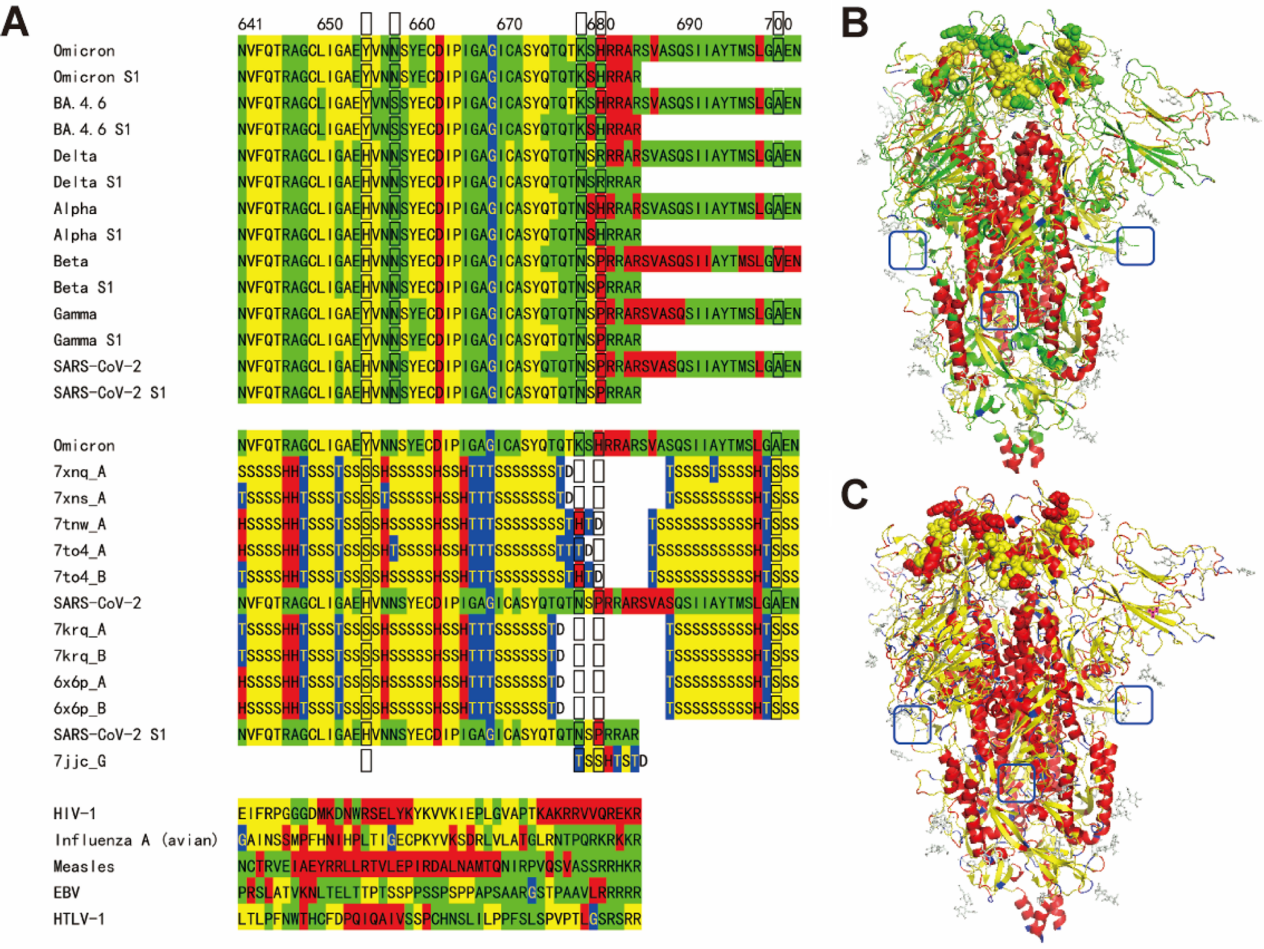
Sequence flexibility/rigidity maps (A) of furin cleavage site for SARS-CoV-2 variants, human immunodeficiency virus 1 (HIV-1), influenza A (avian), measles, Epstein–Barr virus (EBV), and human T cell lymphotropic virus 1 (HTLV-1) with supersecondary structure code (SSSC) maps of PDB structures (green: flexible conformation; red: α-helix-type conformation; yellow: β-sheet-type conformation; blue: other-type conformations; and black frame: mutation site). Conformational variability (B) and experimental SSSC maps (C) of spike protein for BA.5 on cryo-EM structure of PDB ID 7xnq with invisible furin cleavage site (blue frame) and YRYRLFR motif (sphere: residues 451–457) in RBD.

Even though the cell entry through the endocytic pathway, the S1/S2 separation is a prerequisite for membrane fusion. Neuropilin-1 (NRP1) is another host factor that facilitates SARS-CoV-2 infection^20,21^ and has been identified as a direct cargo sorted by the endosomal SNX-BAR sorting complex promoting exit 1 (ESCPE-1) for the endosomal sorting related to the retrograde transport from endosomes to the trans-Golgi network.^22^ NRP1 interacts with the C-end rule (CendR) peptide motif at the furin cleavage site (residues 682–685), but unfortunately, the cryo-EM structures of CendR for Omicron variants remain elusive (Figure 1).

In general, a variety of other-type conformations (“T” conformations), which amino acids in sequences of protein subunits form, apart from an α-helix-type conformation (“H” conformation) and a β-sheet-type conformation (“S” conformation) afford a backbone instability and are supported by electrostatic or hydrogen-bonding interactions in the motif (Figure 1A). The predicted flexibility/rigidity map near the furin cleavage site (residues 641–703) shows the reasonable flexible sites (green highlight) related to the “T” conformations or the invisible sites (Figure 1A). The cleavage sites for cathepsin G and neutrophil elastase near the CendR peptide well correspond to the predicted flexible sites.^23^ The flexibility/rigidity map pattern of CendR for Omicron variants before the S1/S2 cleavage is different from those for Delta and Alpha variants due to the N679K mutation. More importantly, after the cleavage of S1 and S2, only the predicted C-terminus of S1 for Omicron variants is rigid and has the successive “H” conformations similar to the human immunodeficiency virus type 1 (HIV-1) envelope glycoprotein gp160 precursor (Figure 1A). It has been reported that even though short gp160 peptides are efficiently cleaved *in vitro* by furin at Arg^511^, the full length gp160 was shown not to be efficiently cleaved *ex vivo*.^24^ The short CendR peptide of Omicron variant also indicates the more increased intrinsic cleavability with furin than those of Delta and Alpha variants.^25^ Further, though upstream of the physiological processing site, a second *site2* potential furin motif (KAKR^503^) of HIV-1 is inefficiently cleaved,^24^ the flexibility/rigidity map pattern of HIV-1 indicates that the KAKRRVVQREKR motif is also rigid (Figure 1A). The N658S mutation of BA.4.6 changes the wide range pattern of flexibility/rigidity map and makes the CendR more rigid after the cleavage of S1 and S2. This rigidity of CendR for Omicron variants increases the activation energy for the cleavage, and the conformation of CendR for Omicron variants as well as cellular and/or extracellular factors, also influence the efficacy and selectivity of the furin mediated cleavage.^24^ Actually, substrate-like peptide inhibitor Arg-Arg-Arg-Val-Arg-Amba with the interactions of P4–P6 of furin on the protein data bank (PDB)^26^ structure (PDB ID 6eqx) has the SSSC sequence “THSSTD”, which is largely different from the successive “H” conformations of Omicron variants.^27^ Further, as shown below, the successive “H” conformation is seen in the fusion peptide such as SFIEDLLF (residues 816–823) of S2, which has been experimentally confirmed in the antibody complexes,^28^ and the rigidity of CendR for Omicron variants with the successive “H” conformations may contribute to the relatively effective cell entry and the low rate of syncytia formation with Omicron infection regardless of the relative high reproduction.^13,15^

### Receptor Binding Domain and S2 Subunit of Spike Protein

**Figure 2:**
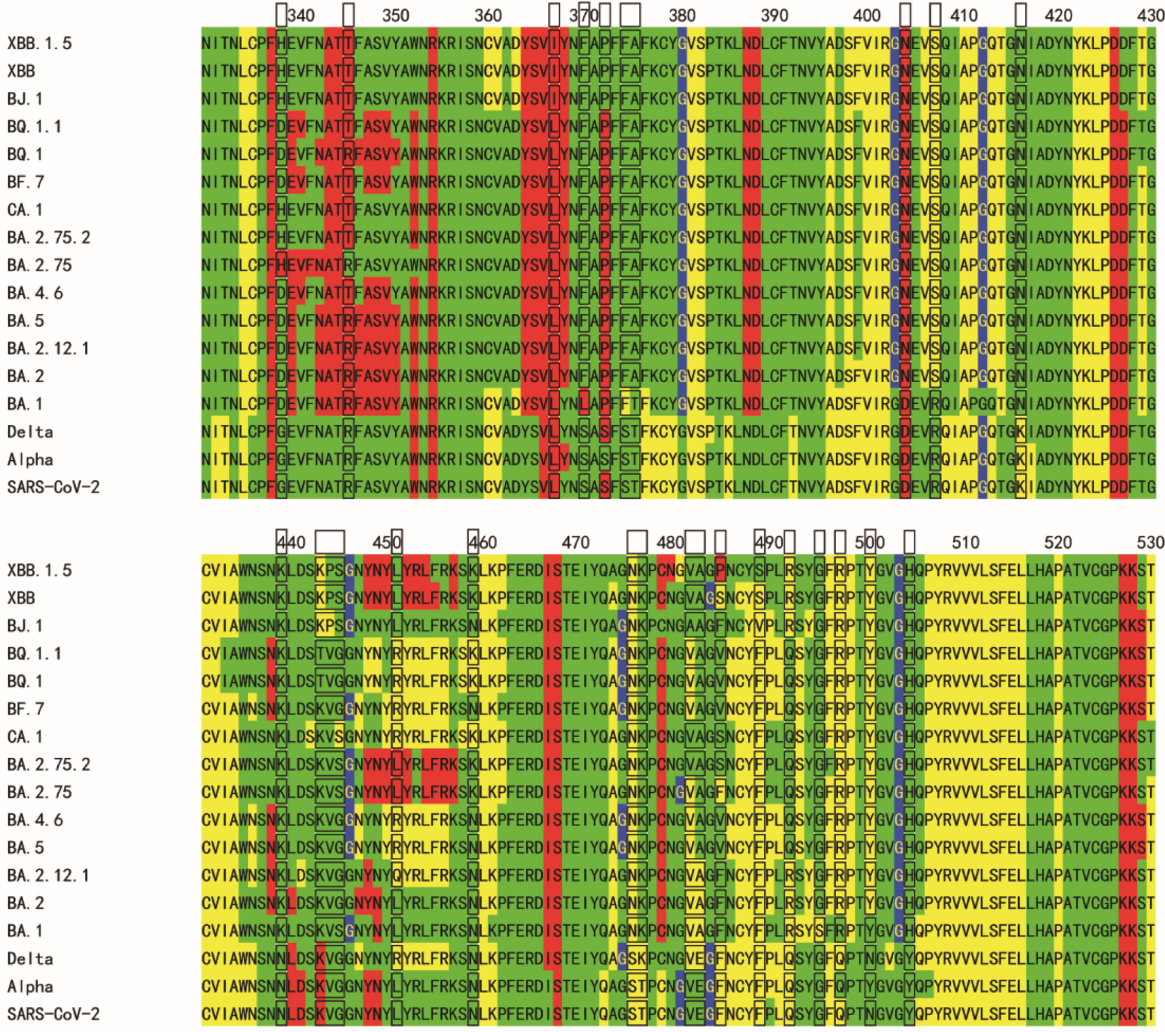
Sequence flexibility/rigidity maps of RBD for SARS-CoV-2 variants (green: flexible conformation; red: α-helix-type conformation; yellow: β-sheet-type conformation; blue: other-type conformations; and black frame: mutation site).

In the RBD region, two large differences (residues 343–351 and residues 449–458) between Omicron variants and the other variants have been predicted (Figure 2). One is similar to Delta variant,^7^ and the L452R mutation increases the rigidity of YRYRLFR motif (residues 451–457) for Omicron variants BA.5, BA.2.12.1, BA.4.6, CA.1, BF.7, BQ.1, and BQ.1.1 with the successive “S” conformations (Figure 1B), which largely corresponds to the cryo-EM structure of PDB ID 7xnq (Figures 1C and S1). The increased rigidity of the edge of flexible regions can afford the thermal stability of Omicron variants, so that it supports the resistance of summer heat. Interestingly, the N460K mutation of BA.2.75 stabilizes the variant in a different way and makes the YNYLYRLFRK motif (residues 449–458) rigid with two successive “H” conformations (Figures 2 and S1). The thermal stability of BA.2.75 due to this N460K mutation is consistent with the resistance of heat in India. Omicron variant XBB, which possesses two successive “H” conformations like BA.2.75, has spread in relatively warm regions such as Singapore.^29^ On the other hand, the K444T mutation of BQ.1.1 and BQ.1^19^ has increased the flexibility of the IAWNSNKLDSTVGGN motif for winter (Figure 2). Further, the F486P mutation of XBB.1.5^19^ makes the NCYSPL motif flexible and contributes to the sharp spreading in United States.

Another predicted large difference is associated with the NATRFASVY motif of Omicron variants (residues 343–351), which does not directly contact to the host receptor ACE2. In this region, the experimental deep mutational scanning of WT has not indicated the clear correlation between lower ACE2-binding affinity and lower expression.^30^ Only the lower expression has suggested some important structural functions of WT. The G339D mutation of Omicron variants increases the rigidity of NATRFASVY motif with the successive “H” conformations, which is different from the cryo-EM structure of PDB ID 7xnq (Figures 2 and S1). For the S1/S2 separation, the conformational change of S1 and S2 is also a prerequisite for membrane fusion, and the G339D mutation can contributes to accelerate this conformational change. Actually, the cryo-EM structure of PDB ID 7xnq suggests the instability of this motif due to the G339D mutation. The D339H mutation of BA.2.75 stabilizes the FHEVFNAT motif (residues 338–345) to largely corresponds to the cryo-EM structures of Omicron variants (Figures 2 and S1). It may be related to the resistance of heat in India. On the other hand, the R346T mutation of BA.4.6, BA.2.75.2, CA.1, BF.7, BQ1.1, BJ.1, XBB, and XBB.1.5 gives further different flexibility to this motif and seems to proceed the optimization of this region after the G339D mutation of initial Omicron variants (Figure 2).

**Figure 3:**
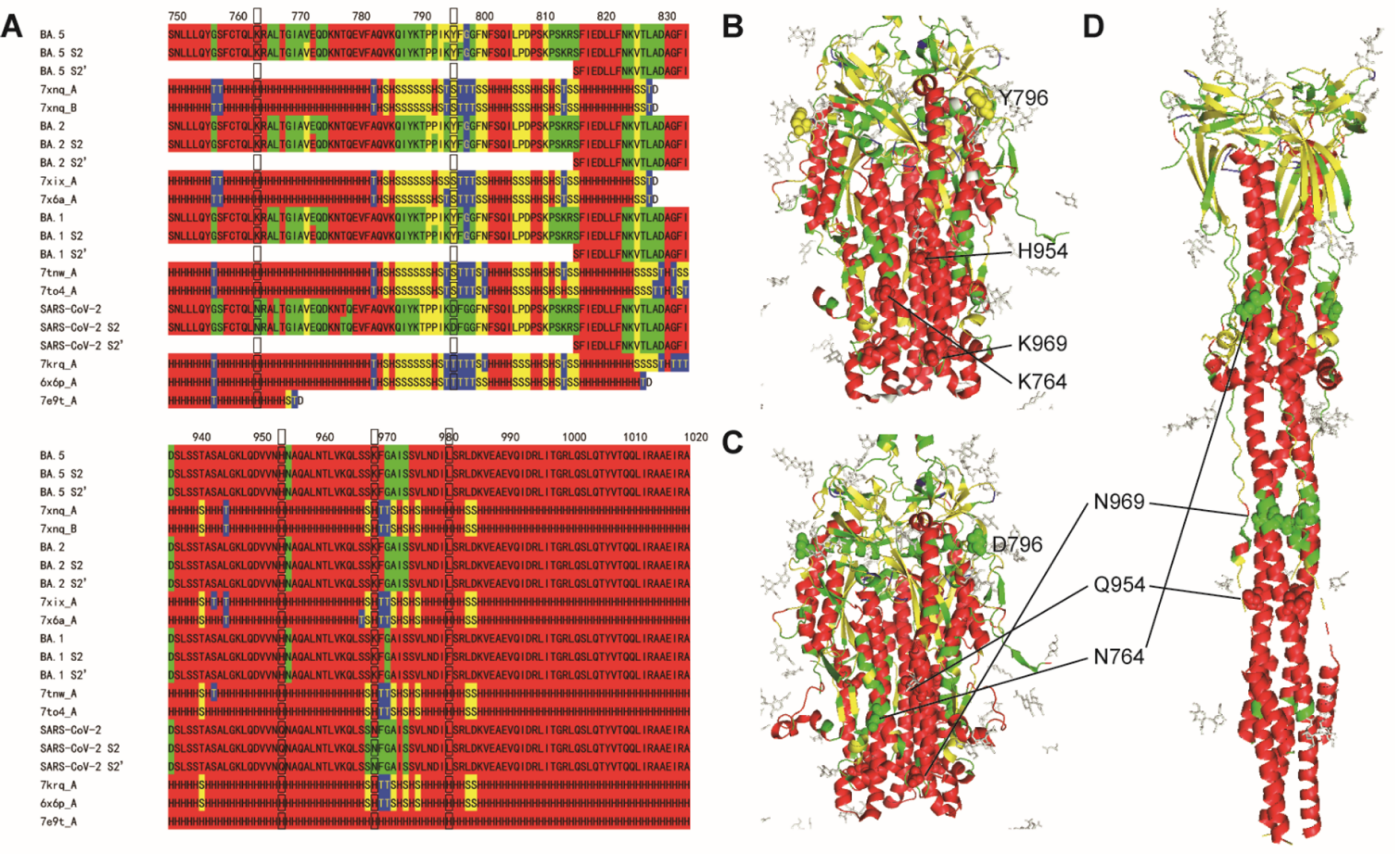
Sequence flexibility/rigidity maps (A) of four common mutation sites in S2 (N764K, D796Y, Q954H, and N969K) for SARS-CoV-2 variants with SSSC maps of PDB structures (green: flexible conformation; red: α-helix-type conformation; yellow: β-sheet-type conformation; blue: other-type conformations; and black frame: mutation site). Conformational variability maps of S2 for BA.5 prefusion state on PDB ID 7xnq (B), for WT prefusion state on PDB ID 7krq (C), and for WT postfusion state on PDB ID 7e9t (D) with common mutation sites (sphere). The S1 subunit is omitted for clarity.

The Omicron variants contain four common mutations in S2 (N764K, D796Y, Q954H, and N969K). The predicted flexibility/rigidity maps near the four mutations (residues 750–834 and residues 936–1020) also demonstrate the reasonable flexible sites (green highlight) related to the “T” conformations or the invisible sites for the conformational change with membrane fusion (Figure 3). For example, the flexible site near the N764K mutation corresponds to the invisible sites for the cryo-EM postfusion structure of PDB ID 7e9t (Figure 3A). As mentioned above, the SFIEDLLF motif (residues 816–823) of fusion peptide, which is also invisible after the cleavage of S2′, has the predicted successive “H” conformations (Figure 3A). The predicted change of conformational variability for these four mutations, slightly affording stability or flexibility of the nearest sites, are very small.

### Thermal Stability of BA.5

**Figure 4:**
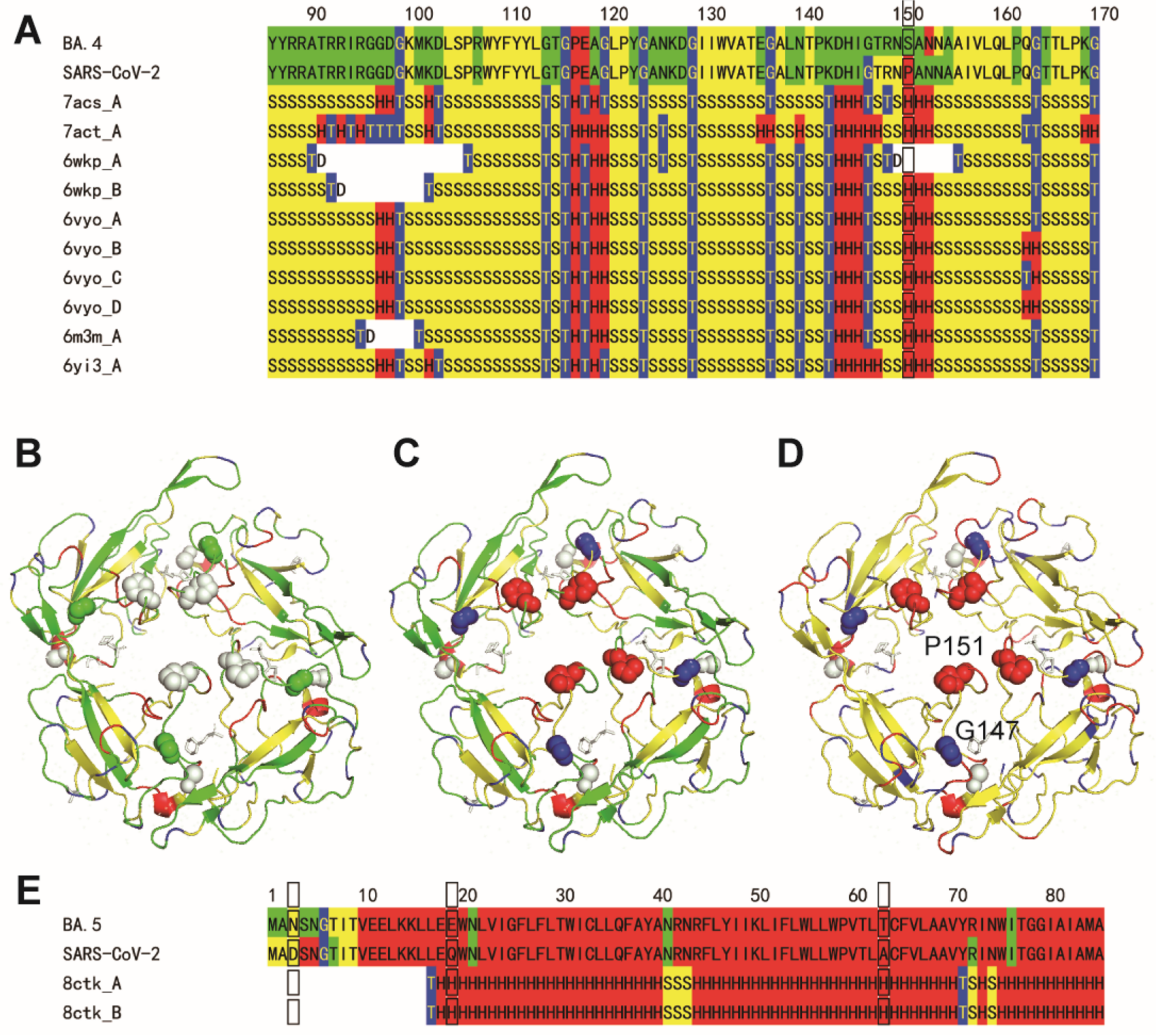
Sequence flexibility/rigidity maps (A) of N proteins for BA.4 and WT near P151S mutation sites with SSSC maps of PDB structures (green: flexible conformation; red: α-helix-type conformation; yellow: β-sheet-type conformation; blue: other-type conformations; and black frame: mutation site). Conformational variability maps of N proteins for BA.4 (B) and WT (C) on PDB ID 6vyo with experimental SSSC map for WT (D) on PDB ID 6vyo with P151 and G147 (sphere). Sequence flexibility/rigidity maps (E) of M proteins for BA.5 and WT near D3N mutation sites with SSSC maps of PDB structures.

BA.5 and BA.4 share many mutations/deletions except BA.5 has the following mutations: membrane (M) glycoprotein: D3N; open reading frame (ORF) 7b: L11 (WT); nucleocapsid (N) phosphoprotein: P151 (WT). Though the frequencies of BA.5 and BA.4 were similar in May 2022, that of BA.5 has been rapidly increased over the summer.^29^ The predicted flexibility/rigidity map near P151S mutation (residues 86–170) also indicates the reasonable flexible sites (green highlight) related to the “T” conformations or the invisible sites (Figure 4). The P151S mutation of N protein for BA.4 makes the GTRNS motif (residues 147–151) including the “T” conformation of glycine flexible (Figure 4A–B). The more rigid motif of BA.5, which corresponds to the structure of PDB ID 6vyo, thermodynamically stabilizes the conformation (Figure 4C–D). On the other hand, though the D3N mutation of M protein for BA.5 forces the neighbor to be flexible, the position has not been determined in the structure of PDB ID 8ctk because of the N terminal (Figure 4E). ORF 7b is also a short peptide, and totally, the thermal stability of BA.5 is suggested as one of the factors for the selection of BA.5 versus BA.4.

SSSCpreds results never definitely explain what we observe from the variants. The results could potentially provide explanations but SSSCpreds or any other computational or experimental tool is not entirely perfect. In future, we aim to construct the deep neural network-based prediction system of correlations between characteristic SSSC patterns and functions related to protein–protein, protein–RNA, and protein–substrate interaction for various spectroscopies including vibrational optical activity spectroscopy.

## Conclusions

In conclusion, the deep neural network-based prediction of conformational variability demonstrates the reasonable flexible sites related to the “T” conformations or the invisible sites of the PDB structures. The less efficient S1/S2 cleavage of Omicron BA.1 variant can be rationalized by the increased activation energy for the cleavage due to the rigidity of CendR for Omicron variants with the successive “H” conformations after the cleavage of S1 and S2. The conformational stability from deep neural network-based prediction is consistent with the thermodynamic stability of variants and potentially explains the difference of seasonable pandemic variants in summer and those in winter. Further, the geographical optimization of variants is also traceable from the conformational variability map.

Combining conformational stability with the thermal environment experienced by the virus variants gives a novel concept and adds a new tool, beyond structure, to understand relative infection ability and ease of spread for the virus. This deep neural network-based prediction method shows its true value only with the structural data such as the cryo-EM structures or AlphaFold2. The accuracy of conformational variability prediction is enough to complement transformation information on motifs in protein structures to AlphaFold2.

## Computational Methods

### SSSCview and SSSCPreds^6^

Supersecondary structure code (SSSC) is expressed as a conformation term for each amino acid peptide unit using the letters H, S, T, and D referring to an α-helix-type conformation (H), a β-sheet-type conformation (S), a variety of other-type conformations (T), and disordered residues or the C-terminus (D), which is derived from the template patterns, represented as conformational codes,^31,32^ such as 3a5c4a (α-helix-type conformation) and 6c4a4a (β-sheet-type conformation).^33^ The observed PDB structure files were converted to the FASTA-format files containing the amino acid sequences and SSSCs of protein subunits using SSSCview.^6,34^ Python^35^ and Biopython^36^ were used to construct SSSCview. Assignment of “T” conformations was carried out using template patterns such as 2c6a4c (left-handed α-helix-type conformation).

The predicted SSSCs were obtained from the FASTA-format files containing the original and mutation sequences of protein subunits using SSSCPreds, which has the benchmarks (average concordance rates) of the three systems SSSCPred200, SSSCPred100, and SSSCPred as follows: for SSSCPred200, CullPDB,^37^ 0.905 (9851 subunits) and CB513,^37^ 0.911 (361 subunits); for SSSCPred100, CullPDB, 0.896 (17,169 subunits) and CB513, 0.907 (612 subunits); and for SSSCPred, CullPDB, 0.861 (17,169 subunits) and CB513, 0.882 (612 subunits).^6^ Python^35^ and Neural Network Console 1.40^38^ were utilized to construct SSSCPreds. Software SSSCPreds and SSSCview have been deposited on H.I. Web site (https://staff.aist.go.jp/izumi.h/SSSCPreds/index-e.html) and have been freely available.

### Conformational Variability Map

Conformational variability for each amino acid peptide unit was judged by using the concordance of SSSCs among SSSCPred200, SSSCPred100, and SSSCPred as the following conformations, a flexible conformation (green), an α-helix-type conformation (red), a β-sheet-type conformation (yellow), and a variety of other-type conformations (blue). Sequence flexibility/rigidity map was constructed using the python script with python-docx.^39^ Conformational variability map on molecular model was created using the python script with PyMOL.^40^

The amino acid sequences of SARS-CoV-2, human immunodeficiency virus 1 (HIV-1), influenza A (avian), measles, Epstein–Barr virus (EBV), and human T cell lymphotropic virus 1 (HTLV-1) were obtained from UniProt.^41^

## Supporting Information Available

Conformational variability maps of RBD with SSSC maps of PDB structures, SSSCPreds data of SARS-CoV-2 variants, HIV-1, influenza A (avian), measles, EBV, and HTLV-1, and SSSC data of PDB structures (PDF)
