# Supplementary material for: Effect of Conformational Variability on Seasonable Thermal Stability and Cell Entry of Omicron Variants
Source: ACS Omega. 2023 Feb 10;8(7):7111–8. doi: 10.1021/acsomega.2c08075 (PMC9948215; doi:10.1021/acsomega.2c08075)
Supplement: Supplementary file 1 — ao2c08075_si_001.pdf [file ao2c08075_si_001.pdf]

Supporting Information for

Effect of conformational variability on seasonable thermal stability and cell entry of Omicron variants

Hiroshi Izumi,[*,†] *Hiroshi Aoki,*[†] *Laurence A. Nafie,*[‡,§] *and Rina K. Dukor*[§]

[†]National Institute of Advanced Industrial Science and Technology (AIST), AIST Tsukuba West, 16-1 Onogawa, Tsukuba, Ibaraki 305-8569, Japan
[‡]Department of Chemistry, Syracuse University, Syracuse, New York 13244-4100, United States
[§]BioTools Inc., Bee Line Hwy, Jupiter, Florida 33458, United States

e-mail: izumi.h@aist.go.jp

Table of contents

Figure S1 S2
Table S1 S3-S9
Table S2 S10-S16

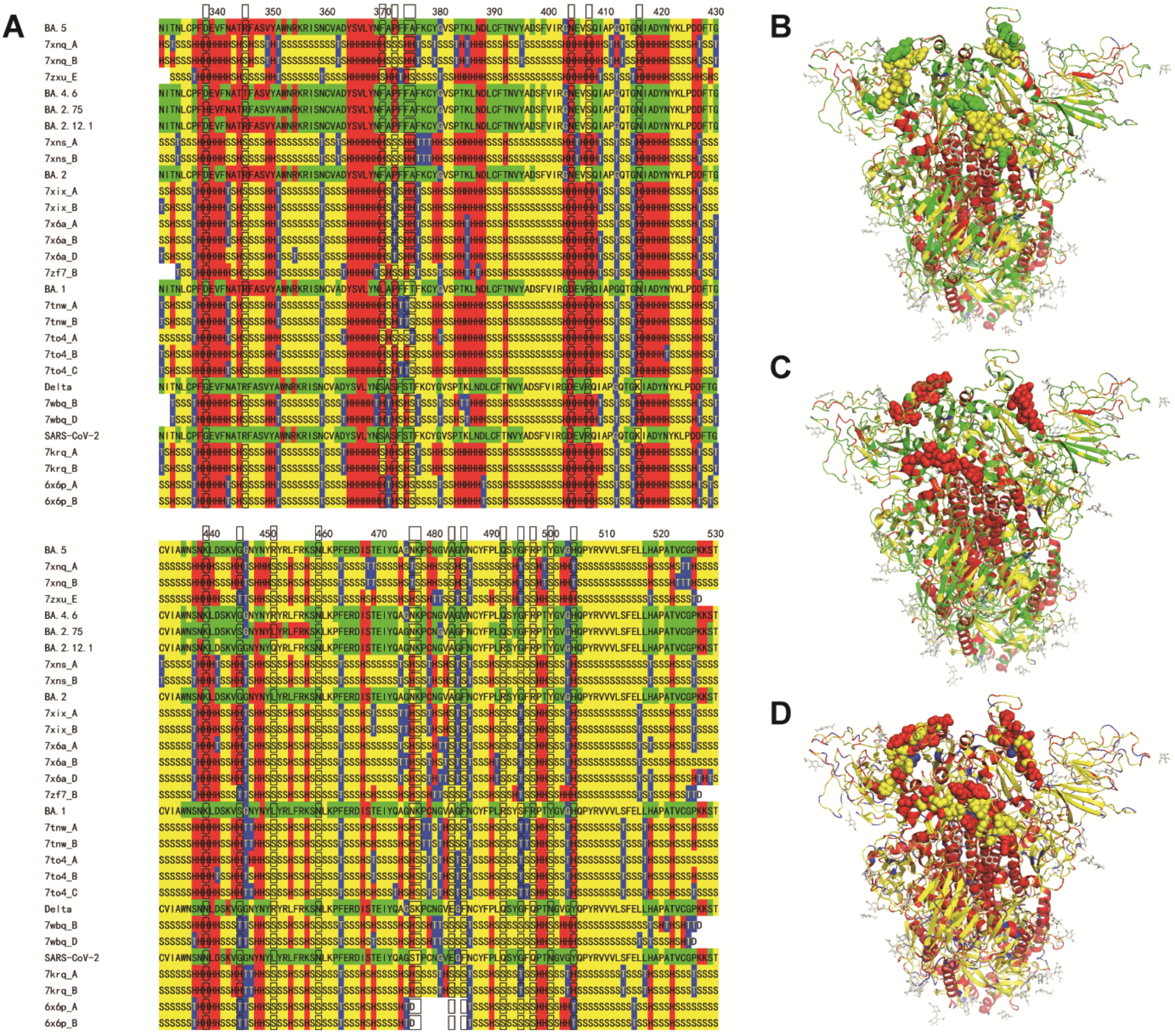

Figure S1. Sequence flexibility/rigidity maps (A) of RBD for SARS-CoV-2 variants with supersecondary structure code (SSSC) maps of PDB structures (green: flexible conformation; red: α-helix-type conformation; yellow: β-sheet-type conformation; blue: other-type conformations; and black frame: mutation site). Conformational variability maps of YRYRLFR motif (B) and NATRFASVY motif (C) for BA.5 on PDB ID 7xnq (sphere) with experimental SSSC map of YRYRLFR and NATRFASVY motifs (D) on PDB ID 7xnq (sphere).

Table S1. SSSCPreds data of SARS-CoV-2 variants, human immunodeficiency virus 1 (HIV-1), influenza A (avian), measles, Epstein Barr virus (EBV), and human T cell lymphotropic virus 1 (HTLV-1).

XBB S

MFVFLVLLPLVSSQCVNLITRTQSYTNSFTRGVYYPDKVFRSSVLHSTQDLFLPFFSNVTWFHAIHVSGTNGTKRFDNPALPFNDGVYFASTEKSNIIRGWIFGTTLDSKT
QSLLIVNNATNVVIKVCEFQFCNDPFLDVYQKNNKSWMESEFRVYSSANNCTFEYVSQPFLMDLEGKEGNFKNLREFVFKNIDGYFKIYSKHTPINLERDLPQGFSALEPLVDLPIGINIT
RFQTLLALHRSYLTPVDSSSGWTAGAAAYYVGYLQPRTFLLKYNENGTITDAVDCALDPLSETKCTLKSFTVEKGIYQTSNFRVQPTESIVRFPNITNLCPFHEVFNATTFASVYAWNRKR
ISNCVADYSVIYNFAPFFAFKCYGVSPTKLNDLCFTNVYADSFVIRGNEVSQIAPGQTGNIADYNYKLPDDFTGCVIAWNSNKLDSKPSGNYNYLYRLFRKSKLKPFERDISTEIYQAGNK
PCNGVAGSNCYSPLRSYGFRPTYGVGHQPYRVVVLSFELLHAPATVCGPKKSTNLVKNKCVNFNFNGLTGTGVLTESNKKFLPFQQFGRDIADTTDAVRDPQTLEILDITPCSFGGVSVIT
PGTNTSNQVAVLYQGVNCTEVPVAIHADQLTPTWRVYSTGSNVFQTRAGCLIGAEYVNNSYECDIPIGAGICASYQTQTKSHRRARSVASQSIIAYTMSLGAENSVAYSNNSIAIPTNFTI
SVTTEILPVSMTKTSVDCTMYICGDSTECSNLLLQYGSFCTQLKRALTGIAVEQDKNTQEVFAQVKQIYKTPPIKYFGGFNFSQILPDPSKPSKRSFIEDLLFNKVTLADAGFIKQYGDCL
GDIAARDLICAQKFNGLTVLPPLLTDEMIAQYTSALLAGTITSGWTFGAGAALQIPFAMQMAYRFNGIGVTQNVLYENQKLIANQFNSAIGKIQDSLSSTASALGKLQDVVNHNAQALNTL
VKQLSSKFGAISSVLNDILSRLDKVEAEVQIDRLITGRLQSLQTYVTQQLIRAAEIRASANLAATKMSECVLGQSKRVDFCGKGYHLMSFPQSAPHGVVFLHVTYVPAQEKNFTTAPAICH
DGKAHFPREGVFVSNGTHWFVTQRNFYEPQIITTDNTFVSGNCDVVIGIVNNTVYDPLQPELDSFKEELDKYFKNHTSPDVDLGDISGINASVVNIQKEIDRLNEVAKNLNESLIDLQELG
KYEQYIKWPWYIWLGFIAGLIAIVMVTIMLCCMTSCCSCLKGCCSCGSCCKFDEDDSEPVLKGVKLHYT

BJ.1 S

MFVFLVLLPLVSSQCVNLITRTQSYTNSFTRGVYYPDKVFRSSVLHSTQDLFLPFFSNVTWFHAIHVSGTNGTKRFDNPALPFNDGVYFASTEKSNIIRGWIFGTTLDSKT
QSLLIVNNATNVVIKVCEFQFCNDPFLDVYQKNNKSWMESEFRVYSSANNCTFEYVSQPFLMDLEGKEGNFKNLREFVFKNIDGYFKIYSKHTPINLERDLPQGFSALEPLVDLPIGINIT
RFQTLLALHRSYLTPGDSSSGWTAGAAAYYVGYLQPRTFLLKYNENGTITDAVDCALDPLSETKCTLKSFTVEKGIYQTSNFRVQPTESIVRFPNITNLCPFHEVFNATTFASVYAWNRKR
ISNCVADYSVIYNFAPFFAFKCYGVSPTKLNDLCFTNVYADSFVIRGNEVSQIAPGQTGNIADYNYKLPDDFTGCVIAWNSNKLDSKPSGNYNYLYRLFRKSNLKPFERDISTEIYQAGNK
PCNGAAGFNCYVPLRSYGFRPTYGVGHQPYRVVVLSFELLHAPATVCGPKKSTNLVKNKCVNFNFNGLTGTGVLTESNKKFLPFQQFGRDIADTTDAVRDPQTLEILDITPCSFGGVSVIT
PGTNTSNQVAVLYQGVNCTEVPVAIHADQLTPTWRVYSTGSNVFQTRAGCLIGAEYVNNSYECDIPIGAGICASYQTQTKSHRRARSVASQSIIAYTMSLGAENSVAYSNNSIAIPTNFTI
SVTTEILPVSMTKTSVDCTMYICGDSTECSNLLLQYGSFCTQLKRALTGIAVEQDKNTQEVFAQVKQIYKTPPIKYFDGFNFSQILPDPSKPSKRSFIEDLLFNKVTLADAGFIKQYGDCL
GDIAARDLICAQKFNGLTVLPPLLTDEMIAQYTSALLAGTITSGWTFGAGAALQIPFAMQMAYRFNGIGVTQNVLYENQKLIANQFNSAIGKIQDSLSSTASALGKLQDVVNHNAQALNTL
VKQLSSKFGAISSVLNDILSRLDKVEAEVQIDRLITGRLQILQTYVTQQLIRAAEIRASANLAATKMSECVLGQSKRVDFCGKGYHLMSFPQSAPHGVVFLHVTYVPAQEKNFTTAPAICH
DGKAHFPREGVFVSNGTHWFVTQRNFYEPQIITTDNTFVSGNCDVVIGIVNNTVYDPLQPELDSFKEELDKYFKNHTSPDVDLGDISGINASVVNIQKEIDRLNEVAKNLNESLIDLQELG
KYEQYIKWPWYIWLGFIAGLIAIVMVTIMLCCMTSCCSCLKGCCSCGSCCKFDEDDSEPVLKGVKLHYT

BQ.1.1 S

MFVFLVLLPLVSSQCVNLITRTQSYTNSFTRGVYYPDKVFRSSVLHSTQDLFLPFFSNVTWFHAISGTNGTKRFDNPVLPFNDGVYFASTEKSNIIRGWIFGTTLDSKTQS
LLIVNNATNVVIKVCEFQFCNDPFLDVYYHKNNKSWMESEFRVYSSANNCTFEYVSQPFLMDLEGKQGNFKNLREFVFKNIDGYFKIYSKHTPINLGRDLPQGFSALEPLVDLPIGINITR
FQTLLALHRSYLTPGDSSSGWTAGAAAYYVGYLQPRTFLLKYNENGTITDAVDCALDPLSETKCTLKSFTVEKGIYQTSNFRVQPTESIVRFPNITNLCPFDEVFNATTFASVYAWNRKRI
SNCVADYSVLYNFAPFFAFKCYGVSPTKLNDLCFTNVYADSFVIRGNEVSQIAPGQTGNIADYNYKLPDDFTGCVIAWNSNKLDSTVGGNYNYRYRLFRKSKLKPFERDISTEIYQAGNKP
CNGVAGVNCYFPLQSYGFRPTYGVGHQPYRVVVLSFELLHAPATVCGPKKSTNLVKNKCVNFNFNGLTGTGVLTESNKKFLPFQQFGRDIADTTDAVRDPQTLEILDITPCSFGGVSVITP
GTNTSNQVAVLYQGVNCTEVPVAIHADQLTPTWRVYSTGSNVFQTRAGCLIGAEYVNNSYECDIPIGAGICASYQTQTKSHRRARSVASQSIIAYTMSLGAENSVAYSNNSIAIPTNFTIS
VTTEILPVSMTKTSVDCTMYICGDSTECSNLLLQYGSFCTQLKRALTGIAVEQDKNTQEVFAQVKQIYKTPPIKYFGGFNFSQILPDPSKPSKRSFIEDLLFNKVTLADAGFIKQYGDCLG
DIAARDLICAQKFNGLTVLPPLLTDEMIAQYTSALLAGTITSGWTFGAGAALQIPFAMQMAYRFNGIGVTQNVLYENQKLIANQFNSAIGKIQDSLSSTASALGKLQDVVNHNAQALNTLV
KQLSSKFGAISSVLNDILSRLDKVEAEVQIDRLITGRLQSLQTYVTQQLIRAAEIRASANLAATKMSECVLGQSKRVDFCGKGYHLMSFPQSAPHGVVFLHVTYVPAQEKNFTTAPAICHD
GKAHFPREGVFVSNGTHWFVTQRNFYEPQIITTDNTFVSGNCDVVIGIVNNTVYDPLQPELDSFKEELDKYFKNHTSPDVDLGDISGINASVVNIQKEIDRLNEVAKNLNESLIDLQELGK
YEQYIKWPWYIWLGFIAGLIAIVMVTIMLCCMTSCCSCLKGCCSCGSCCKFDEDDSEPVLKGVKLHYT

BQ.1 S

MFVFLVLLPLVSSQCVNLITRTQSYTNSFTRGVYYPDKVFRSSVLHSTQDLFLPFFSNVTWFHAISGTNGTKRFDNPVLPFNDGVYFASTEKSNIIRGWIFGTTLDSKTQS
LLIVNNATNVVIKVCEFQFCNDPFLDVYYHKNNKSWMESEFRVYSSANNCTFEYVSQPFLMDLEGKQGNFKNLREFVFKNIDGYFKIYSKHTPINLGRDLPQGFSALEPLVDLPIGINITR
FQTLLALHRSYLTPGDSSSGWTAGAAAYYVGYLQPRTFLLKYNENGTITDAVDCALDPLSETKCTLKSFTVEKGIYQTSNFRVQPTESIVRFPNITNLCPFDEVFNATRFASVYAWNRKRI
SNCVADYSVLYNFAPFFAFKCYGVSPTKLNDLCFTNVYADSFVIRGNEVSQIAPGQTGNIADYNYKLPDDFTGCVIAWNSNKLDSTVGGNYNYRYRLFRKSKLKPFERDISTEIYQAGNKP
CNGVAGVNCYFPLQSYGFRPTYGVGHQPYRVVVLSFELLHAPATVCGPKKSTNLVKNKCVNFNFNGLTGTGVLTESNKKFLPFQQFGRDIADTTDAVRDPQTLEILDITPCSFGGVSVITP
GTNTSNQVAVLYQGVNCTEVPVAIHADQLTPTWRVYSTGSNVFQTRAGCLIGAEYVNNSYECDIPIGAGICASYQTQTKSHRRARSVASQSIIAYTMSLGAENSVAYSNNSIAIPTNFTIS
VTTEILPVSMTKTSVDCTMYICGDSTECSNLLLQYGSFCTQLKRALTGIAVEQDKNTQEVFAQVKQIYKTPPIKYFGGFNFSQILPDPSKPSKRSFIEDLLFNKVTLADAGFIKQYGDCLG
DIAARDLICAQKFNGLTVLPPLLTDEMIAQYTSALLAGTITSGWTFGAGAALQIPFAMQMAYRFNGIGVTQNVLYENQKLIANQFNSAIGKIQDSLSSTASALGKLQDVVNHNAQALNTLV
KQLSSKFGAISSVLNDILSRLDKVEAEVQIDRLITGRLQSLQTYVTQQLIRAAEIRASANLAATKMSECVLGQSKRVDFCGKGYHLMSFPQSAPHGVVFLHVTYVPAQEKNFTTAPAICHD
GKAHFPREGVFVSNGTHWFVTQRNFYEPQIITTDNTFVSGNCDVVIGIVNNTVYDPLQPELDSFKEELDKYFKNHTSPDVDLGDISGINASVVNIQKEIDRLNEVAKNLNESLIDLQELGK
YEQYIKWPWYIWLGFIAGLIAIVMVTIMLCCMTSCCSCLKGCCSCGSCCKFDEDDSEPVLKGVKLHYT

BF.7 S

MFVFLVLLPLVSSQCVNLITRTQSYTNSFTRGVYYPDKVFRSSVLHSTQDLFLPFFSNVTWFHAISGTNGTKRFDNPVLPFNDGVYFASTEKSNIIRGWIFGTTLDSKTQS
LLIVNNATNVVIKVCEFQFCNDPFLDVYYHKNNKSWMESEFRVYSSANNCTFEYVSQPFLMDLEGKQGNFKNLREFVFKNIDGYFKIYSKHTPINLGRDLPQGFSALEPLVDLPIGINITR
FQTLLALHRSYLTPGDSSSGWTAGAAAYYVGYLQPRTFLLKYNENGTITDAVDCALDPLSETKCTLKSFTVEKGIYQTSNFRVQPTESIVRFPNITNLCPFDEVFNATTFASVYAWNRKRI
SNCVADYSVLYNFAPFFAFKCYGVSPTKLNDLCFTNVYADSFVIRGNEVSQIAPGQTGNIADYNYKLPDDFTGCVIAWNSNKLDSKVGGNYNYRYRLFRKSNLKPFERDISTEIYQAGNKP
CNGVAGVNCYFPLQSYGFRPTYGVGHQPYRVVVLSFELLHAPATVCGPKKSTNLVKNKCVNFNFNGLTGTGVLTESNKKFLPFQQFGRDIADTTDAVRDPQTLEILDITPCSFGGVSVITP
GTNTSNQVAVLYQGVNCTEVPVAIHADQLTPTWRVYSTGSNVFQTRAGCLIGAEYVNNSYECDIPIGAGICASYQTQTKSHRRARSVASQSIIAYTMSLGAENSVAYSNNSIAIPTNFTIS
VTTEILPVSMTKTSVDCTMYICGDSTECSNLLLQYGSFCTQLKRALTGIAVEQDKNTQEVFAQVKQIYKTPPIKYFGGFNFSQILPDPSKPSKRSFIEDLLFNKVTLADAGFIKQYGDCLG
DIAARDLICAQKFNGLTVLPPLLTDEMIAQYTSALLAGTITSGWTFGAGAALQIPFAMQMAYRFNGIGVTQNVLYENQKLIANQFNSAIGKIQDSLSSTASALGKLQDVVNHNAQALNTLV
KQLSSKFGAISSVLNDILSRLDKVEAEVQIDRLITGRLQSLQTYVTQQLIRAAEIRASANLAATKMSECVLGQSKRVDFCGKGYHLMSFPQSAPHGVVFLHVTYVPAQEKNFTTAPAICHD
GKAHFPREGVFVSNGTHWFVTQRNFYEPQIITTDNTFVSGNCDVVIGIVNNTVYDPLQPELDSFKEELDKYFKNHTSPDVDLGDISGINASVVNIQKEIDRLNEVAKNLNESLIDLQELGK
YEQYIKWPWYIWLGFIAGLIAIVMVTIMLCCMTSCCSCLKGCCSCGSCCKFDEDDSEPVLKGVKLHYT

CA.1 S

MFVFLVLLPLVSSQCVNLITRTQSYTNSFTRGVYYPDKVFRSSVLHSTQDLFLPFFSNVTWFHAIHVSGTNGTKRFDNPVLPFNDGVYFASTEKSNIIRGWIFGTTLDSKT
QSLLIVNNATNVVIKVCEFQFCNDPFLDVYYHENNKSRMESELRVYSSANNCTFEYVSQPFLMDLEGKQGNFKNLREFVFKNIDGYFKIYSKHTPVNLGRDLPQGFSALEPLVDLPIGINI
TRFQTLLALHRSYLTPGDSSSSWTAGAAAYYVGYLQPRTFLLKYNENGTITDAVDCALDPLSETKCTLKSFTVEKGIYQTSNFRVQPTESIVRFPNITNLCPFHEVFNATTFASVYAWNRK
RISNCVADYSVLYNFAPFFAFKCYGVSPTKLNDLCFTNVYADSFVIRGNEVSQIAPGQTGNIADYNYKLPDDFTGCVIAWNSNKLDSKVSGNYNYRYRLFRKSKLKPFERDISTEIYQAGN
KPCNGVAGSNCYFPLQSYGFRPTYGVGHQPYRVVVLSFELLHAPATVCGPKKSTNLVKNKCVNFNFNGLTGTGVLTESNKKFLPFQQFGRDIADTTDAVRDPQTLEILDITPCSFGGVSVI
TPGTNTSNQVAVLYQGVNCTEVPVAIHADQLTPTWRVYSTGSNVFQTRAGCLIGAEYVNNSYECDIPIGAGICASYQTQTKSHRRARSVASQSIIAYTMSLGAENSVAYSNNSIAIPTNFT
ISVTTEILPVSMTKTSVDCTMYICGDSTECSNLLLQYGSFCTQLKRALTGIAVEQDKNTQEVFAQVKQIYKTPPIKYFGGFNFSQILPDPSKPSKRSFIEDLLFNKVTLADAGFIKQYGDC
LGDIAARDLICAQKFNGLTVLPPLLTDEMIAQYTSALLAGTITSGWTFGAGAALQIPFAMQMAYRFNGIGVTQNVLYENQKLIANQFNSAIGKIQDSLSSTASALGKLQDVVNHNAQALNT
LVKQLSSKFGAISSVLNDILSRLDKVEAEVQIDRLITGRLQSLQTYVTQQLIRAAEIRASANLAATKMSECVLGQSKRVDFCGKGYHLMSFPQSAPHGVVFLHVTYVPAQEKNFTTAPAIC
HDGKAHFPREGVFVSNGTHWFVTQRNFYEPQIITTDNTFVSGNCDVVIGIVNNTVYDPLQPELDSFKEELDKYFKNHTSPDVDLGDISGINASVVNIQKEIDRLNEVAKNLNESLIDLQEL
GKYEQYIKWPWYIWLGFIAGLIAIVMVTIMLCCMTSCCSCLKGCCSCGSCCKFDEDDSEPVLKGVKLHYT

BA.2.75.2 S

MFVFLVLLPLVSSQCVNLITRTQSYTNSFTRGVYYPDKVFRSSVLHSTQDLFLPFFSNVTWFHAIHVSGTNGTKRFDNPVLPFNDGVYFASTEKSNIIRGWIFGTTLDSKT
QSLLIVNNATNVVIKVCEFQFCNDPFLDVYYHENNKSRMESELRVYSSANNCTFEYVSQPFLMDLEGKQGNFKNLREFVFKNIDGYFKIYSKHTPVNLGRDLPQGFSALEPLVDLPIGINI
TRFQTLLALHRSYLTPGDSSSSWTAGAAAYYVGYLQPRTFLLKYNENGTITDAVDCALDPLSETKCTLKSFTVEKGIYQTSNFRVQPTESIVRFPNITNLCPFHEVFNATTFASVYAWNRK
RISNCVADYSVLYNFAPFFAFKCYGVSPTKLNDLCFTNVYADSFVIRGNEVSQIAPGQTGNIADYNYKLPDDFTGCVIAWNSNKLDSKVSGNYNYLYRLFRKSKLKPFERDISTEIYQAGN
KPCNGVAGSNCYFPLQSYGFRPTYGVGHQPYRVVVLSFELLHAPATVCGPKKSTNLVKNKCVNFNFNGLTGTGVLTESNKKFLPFQQFGRDIADTTDAVRDPQTLEILDITPCSFGGVSVI
TPGTNTSNQVAVLYQGVNCTEVPVAIHADQLTPTWRVYSTGSNVFQTRAGCLIGAEYVNNSYECDIPIGAGICASYQTQTKSHRRARSVASQSIIAYTMSLGAENSVAYSNNSIAIPTNFT
ISVTTEILPVSMTKTSVDCTMYICGDSTECSNLLLQYGSFCTQLKRALTGIAVEQDKNTQEVFAQVKQIYKTPPIKYFGGFNFSQILPDPSKPSKRSFIEDLLFNKVTLADAGFIKQYGDC
LGDIAARDLICAQKFNGLTVLPPLLTDEMIAQYTSALLAGTITSGWTFGAGAALQIPFAMQMAYRFNGIGVTQNVLYENQKLIANQFNSAIGKIQDSLSSTASALGKLQDVVNHNAQALNT
LVKQLSSKFGAISSVLNDILSRLDKVEAEVQIDRLITGRLQSLQTYVTQQLIRAAEIRASANLAATKMSECVLGQSKRVDFCGKGYHLMSFPQSAPHGVVFLHVTYVPAQEKNFTTAPAIC
HDGKAHFPREGVFVSNGTHWFVTQRNFYEPQIITTDNTFVSGNCDVVIGIVNNTVYDPLQPELDSFKEELDKYFKNHTSPDVDLGDISGINASVVNIQKEIDRLNEVAKNLNESLIDLQEL
GKYEQYIKWPWYIWLGFIAGLIAIVMVTIMLCCMTSCCSCLKGCCSCGSCCKFDEDDSEPVLKGVKLHYT

BA.5 S

MFVFLVLLPLVSSQCVNLITRTQSYTNSFTRGVYYPDKVFRSSVLHSTQDLFLPFFSNVTWFHAISGTNGTKRFDNPVLPFNDGVYFASTEKSNIIRGWIFGTTLDSKTQS
LLIVNNATNVVIKVCEFQFCNDPFLDVYYHKNNKSWMESEFRVYSSANNCTFEYVSQPFLMDLEGKQGNFKNLREFVFKNIDGYFKIYSKHTPINLGRDLPQGFSALEPLVDLPIGINITR
FQTLLALHRSYLTPGDSSSGWTAGAAAYYVGYLQPRTFLLKYNENGTITDAVDCALDPLSETKCTLKSFTVEKGIYQTSNFRVQPTESIVRFPNITNLCPFDEVFNATRFASVYAWNRKRI
SNCVADYSVLYNFAPFFAFKCYGVSPTKLNDLCFTNVYADSFVIRGNEVSQIAPGQTGNIADYNYKLPDDFTGCVIAWNSNKLDSKVGGNYNYRYRLFRKSNLKPFERDISTEIYQAGNKP
CNGVAGVNCYFPLQSYGFRPTYGVGHQPYRVVVLSFELLHAPATVCGPKKSTNLVKNKCVNFNFNGLTGTGVLTESNKKFLPFQQFGRDIADTTDAVRDPQTLEILDITPCSFGGVSVITP
GTNTSNQVAVLYQGVNCTEVPVAIHADQLTPTWRVYSTGSNVFQTRAGCLIGAEYVNNSYECDIPIGAGICASYQTQTKSHRRARSVASQSIIAYTMSLGAENSVAYSNNSIAIPTNFTIS
VTTEILPVSMTKTSVDCTMYICGDSTECSNLLLQYGSFCTQLKRALTGIAVEQDKNTQEVFAQVKQIYKTPPIKYFGGFNFSQILPDPSKPSKRSFIEDLLFNKVTLADAGFIKQYGDCLG
DIAARDLICAQKFNGLTVLPPLLTDEMIAQYTSALLAGTITSGWTFGAGAALQIPFAMQMAYRFNGIGVTQNVLYENQKLIANQFNSAIGKIQDSLSSTASALGKLQDVVNHNAQALNTLV
KQLSSKFGAISSVLNDILSRLDKVEAEVQIDRLITGRLQSLQTYVTQQLIRAAEIRASANLAATKMSECVLGQSKRVDFCGKGYHLMSFPQSAPHGVVFLHVTYVPAQEKNFTTAPAICHD
GKAHFPREGVFVSNGTHWFVTQRNFYEPQIITTDNTFVSGNCDVVIGIVNNTVYDPLQPELDSFKEELDKYFKNHTSPDVDLGDISGINASVVNIQKEIDRLNEVAKNLNESLIDLQELGK
YEQYIKWPWYIWLGFIAGLIAIVMVTIMLCCMTSCCSCLKGCCSCGSCCKFDEDDSEPVLKGVKLHYT

BA.5 S2

SVASQSIIAYTMSLGAENSVAYSNNSIAIPTNFTISVTTEILPVSMTKTSVDCTMYICGDSTECSNLLLQYGSFCTQLKRALTGIAVEQDKNTQEVFAQVKQIYKTPPIKY
FGGFNFSQILPDPSKPSKRSFIEDLLFNKVTLADAGFIKQYGDCLGDIAARDLICAQKFNGLTVLPPLLTDEMIAQYTSALLAGTITSGWTFGAGAALQIPFAMQMAYRFNGIGVTQNVLY
ENQKLIANQFNSAIGKIQDSLSSTASALGKLQDVVNHNAQALNTLVKQLSSKFGAISSVLNDILSRLDKVEAEVQIDRLITGRLQSLQTYVTQQLIRAAEIRASANLAATKMSECVLGQSK
RVDFCGKGYHLMSFPQSAPHGVVFLHVTYVPAQEKNFTTAPAICHDGKAHFPREGVFVSNGTHWFVTQRNFYEPQIITTDNTFVSGNCDVVIGIVNNTVYDPLQPELDSFKEELDKYFKNH
TSPDVDLGDISGINASVVNIQKEIDRLNEVAKNLNESLIDLQELGKYEQYIKWPWYIWLGFIAGLIAIVMVTIMLCCMTSCCSCLKGCCSCGSCCKFDEDDSEPVLKGVKLHYT

BA.5 S2'

SFIEDLLFNKVTLADAGFIKQYGDCLGDIAARDLICAQKFNGLTVLPPLLTDEMIAQYTSALLAGTITSGWTFGAGAALQIPFAMQMAYRFNGIGVTQNVLYENQKLIANQ
FNSAIGKIQDSLSSTASALGKLQDVVNHNAQALNTLVKQLSSKFGAISSVLNDILSRLDKVEAEVQIDRLITGRLQSLQTYVTQQLIRAAEIRASANLAATKMSECVLGQSKRVDFCGKGY
HLMSFPQSAPHGVVFLHVTYVPAQEKNFTTAPAICHDGKAHFPREGVFVSNGTHWFVTQRNFYEPQIITTDNTFVSGNCDVVIGIVNNTVYDPLQPELDSFKEELDKYFKNHTSPDVDLGD
ISGINASVVNIQKEIDRLNEVAKNLNESLIDLQELGKYEQYIKWPWYIWLGFIAGLIAIVMVTIMLCCMTSCCSCLKGCCSCGSCCKFDEDDSEPVLKGVKLHYT

BA.4.6 S

MFVFLVLLPLVSSQCVNLITRTQSYTNSFTRGVYYPDKVFRSSVLHSTQDLFLPFFSNVTWFHAISGTNGTKRFDNPVLPFNDGVYFASTEKSNIIRGWIFGTTLDSKTQS
LLIVNNATNVVIKVCEFQFCNDPFLDVYYHKNNKSWMESEFRVYSSANNCTFEYVSQPFLMDLEGKQGNFKNLREFVFKNIDGYFKIYSKHTPINLGRDLPQGFSALEPLVDLPIGINITR
FQTLLALHRSYLTPGDSSSGWTAGAAAYYVGYLQPRTFLLKYNENGTITDAVDCALDPLSETKCTLKSFTVEKGIYQTSNFRVQPTESIVRFPNITNLCPFDEVFNATTFASVYAWNRKRI
SNCVADYSVLYNFAPFFAFKCYGVSPTKLNDLCFTNVYADSFVIRGNEVSQIAPGQTGNIADYNYKLPDDFTGCVIAWNSNKLDSKVGGNYNYRYRLFRKSNLKPFERDISTEIYQAGNKP
CNGVAGVNCYFPLQSYGFRPTYGVGHQPYRVVVLSFELLHAPATVCGPKKSTNLVKNKCVNFNFNGLTGTGVLTESNKKFLPFQQFGRDIADTTDAVRDPQTLEILDITPCSFGGVSVITP
GTNTSNQVAVLYQGVNCTEVPVAIHADQLTPTWRVYSTGSNVFQTRAGCLIGAEYVNSSYECDIPIGAGICASYQTQTKSHRRARSVASQSIIAYTMSLGAENSVAYSNNSIAIPTNFTIS
VTTEILPVSMTKTSVDCTMYICGDSTECSNLLLQYGSFCTQLKRALTGIAVEQDKNTQEVFAQVKQIYKTPPIKYFGGFNFSQILPDPSKPSKRSFIEDLLFNKVTLADAGFIKQYGDCLG
DIAARDLICAQKFNGLTVLPPLLTDEMIAQYTSALLAGTITSGWTFGAGAALQIPFAMQMAYRFNGIGVTQNVLYENQKLIANQFNSAIGKIQDSLSSTASALGKLQDVVNHNAQALNTLV
KQLSSKFGAISSVLNDILSRLDKVEAEVQIDRLITGRLQSLQTYVTQQLIRAAEIRASANLAATKMSECVLGQSKRVDFCGKGYHLMSFPQSAPHGVVFLHVTYVPAQEKNFTTAPAICHD
GKAHFPREGVFVSNGTHWFVTQRNFYEPQIITTDNTFVSGNCDVVIGIVNNTVYDPLQPELDSFKEELDKYFKNHTSPDVDLGDISGINASVVNIQKEIDRLNEVAKNLNESLIDLQELGK
YEQYIKWPWYIWLGFIAGLIAIVMVTIMLCCMTSCCSCLKGCCSCGSCCKFDEDDSEPVLKGVKLHYT

BA.4.6 S1

MFVFLVLLPLVSSQCVNLITRTQSYTNSFTRGVYYPDKVFRSSVLHSTQDLFLPFFSNVTWFHAISGTNGTKRFDNPVLPFNDGVYFASTEKSNIIRGWIFGTTLDSKTQS
LLIVNNATNVVIKVCEFQFCNDPFLDVYYHKNNKSWMESEFRVYSSANNCTFEYVSQPFLMDLEGKQGNFKNLREFVFKNIDGYFKIYSKHTPINLGRDLPQGFSALEPLVDLPIGINITR
FQTLLALHRSYLTPGDSSSGWTAGAAAYYVGYLQPRTFLLKYNENGTITDAVDCALDPLSETKCTLKSFTVEKGIYQTSNFRVQPTESIVRFPNITNLCPFDEVFNATTFASVYAWNRKRI
SNCVADYSVLYNFAPFFAFKCYGVSPTKLNDLCFTNVYADSFVIRGNEVSQIAPGQTGNIADYNYKLPDDFTGCVIAWNSNKLDSKVGGNYNYRYRLFRKSNLKPFERDISTEIYQAGNKP
CNGVAGVNCYFPLQSYGFRPTYGVGHQPYRVVVLSFELLHAPATVCGPKKSTNLVKNKCVNFNFNGLTGTGVLTESNKKFLPFQQFGRDIADTTDAVRDPQTLEILDITPCSFGGVSVITP
GTNTSNQVAVLYQGVNCTEVPVAIHADQLTPTWRVYSTGSNVFQTRAGCLIGAEYVNSSYECDIPIGAGICASYQTQTKSHRRAR

BA.2.75 S

MFVFLVLLPLVSSQCVNLITRTQSYTNSFTRGVYYPDKVFRSSVLHSTQDLFLPFFSNVTWFHAIHVSGTNGTKRFDNPVLPFNDGVYFASTEKSNIIRGWIFGTTLDSKT
QSLLIVNNATNVVIKVCEFQFCNDPFLDVYYHENNKSRMESELRVYSSANNCTFEYVSQPFLMDLEGKQGNFKNLREFVFKNIDGYFKIYSKHTPVNLGRDLPQGFSALEPLVDLPIGINI
TRFQTLLALHRSYLTPGDSSSSWTAGAAAYYVGYLQPRTFLLKYNENGTITDAVDCALDPLSETKCTLKSFTVEKGIYQTSNFRVQPTESIVRFPNITNLCPFHEVFNATRFASVYAWNRK
RISNCVADYSVLYNFAPFFAFKCYGVSPTKLNDLCFTNVYADSFVIRGNEVSQIAPGQTGNIADYNYKLPDDFTGCVIAWNSNKLDSKVSGNYNYLYRLFRKSKLKPFERDISTEIYQAGN
KPCNGVAGFNCYFPLQSYGFRPTYGVGHQPYRVVVLSFELLHAPATVCGPKKSTNLVKNKCVNFNFNGLTGTGVLTESNKKFLPFQQFGRDIADTTDAVRDPQTLEILDITPCSFGGVSVI
TPGTNTSNQVAVLYQGVNCTEVPVAIHADQLTPTWRVYSTGSNVFQTRAGCLIGAEYVNNSYECDIPIGAGICASYQTQTKSHRRARSVASQSIIAYTMSLGAENSVAYSNNSIAIPTNFT
ISVTTEILPVSMTKTSVDCTMYICGDSTECSNLLLQYGSFCTQLKRALTGIAVEQDKNTQEVFAQVKQIYKTPPIKYFGGFNFSQILPDPSKPSKRSFIEDLLFNKVTLADAGFIKQYGDC
LGDIAARDLICAQKFNGLTVLPPLLTDEMIAQYTSALLAGTITSGWTFGAGAALQIPFAMQMAYRFNGIGVTQNVLYENQKLIANQFNSAIGKIQDSLSSTASALGKLQDVVNHNAQALNT
LVKQLSSKFGAISSVLNDILSRLDKVEAEVQIDRLITGRLQSLQTYVTQQLIRAAEIRASANLAATKMSECVLGQSKRVDFCGKGYHLMSFPQSAPHGVVFLHVTYVPAQEKNFTTAPAIC
HDGKAHFPREGVFVSNGTHWFVTQRNFYEPQIITTDNTFVSGNCDVVIGIVNNTVYDPLQPELDSFKEELDKYFKNHTSPDVDLGDISGINASVVNIQKEIDRLNEVAKNLNESLIDLQEL
GKYEQYIKWPWYIWLGFIAGLIAIVMVTIMLCCMTSCCSCLKGCCSCGSCCKFDEDDSEPVLKGVKLHYT

BA.2.12.1 S

MFVFLVLLPLVSSQCVNLITRTQSYTNSFTRGVYYPDKVFRSSVLHSTQDLFLPFFSNVTWFHAIHVSGTNGTKRFDNPVLPFNDGVYFASTEKSNIIRGWIFGTTLDSKT
QSLLIVNNATNVVIKVCEFQFCNDPFLDVYYHKNNKSWMESEFRVYSSANNCTFEYVSQPFLMDLEGKQGNFKNLREFVFKNIDGYFKIYSKHTPINLGRDLPQGFSALEPLVDLPIGINI
TRFQTLLALHRSYLTPGDSSSGWTAGAAAYYVGYLQPRTFLLKYNENGTITDAVDCALDPLSETKCTLKSFTVEKGIYQTSNFRVQPTESIVRFPNITNLCPFDEVFNATRFASVYAWNRK
RISNCVADYSVLYNFAPFFAFKCYGVSPTKLNDLCFTNVYADSFVIRGNEVSQIAPGQTGNIADYNYKLPDDFTGCVIAWNSNKLDSKVGGNYNYQYRLFRKSNLKPFERDISTEIYQAGN
KPCNGVAGFNCYFPLRSYGFRPTYGVGHQPYRVVVLSFELLHAPATVCGPKKSTNLVKNKCVNFNFNGLTGTGVLTESNKKFLPFQQFGRDIADTTDAVRDPQTLEILDITPCSFGGVSVI
TPGTNTSNQVAVLYQGVNCTEVPVAIHADQLTPTWRVYSTGSNVFQTRAGCLIGAEYVNNSYECDIPIGAGICASYQTQTKSHRRARSVASQSIIAYTMSLGAENLVAYSNNSIAIPTNFT
ISVTTEILPVSMTKTSVDCTMYICGDSTECSNLLLQYGSFCTQLKRALTGIAVEQDKNTQEVFAQVKQIYKTPPIKYFGGFNFSQILPDPSKPSKRSFIEDLLFNKVTLADAGFIKQYGDC
LGDIAARDLICAQKFNGLTVLPPLLTDEMIAQYTSALLAGTITSGWTFGAGAALQIPFAMQMAYRFNGIGVTQNVLYENQKLIANQFNSAIGKIQDSLSSTASALGKLQDVVNHNAQALNT
LVKQLSSKFGAISSVLNDILSRLDKVEAEVQIDRLITGRLQSLQTYVTQQLIRAAEIRASANLAATKMSECVLGQSKRVDFCGKGYHLMSFPQSAPHGVVFLHVTYVPAQEKNFTTAPAIC
HDGKAHFPREGVFVSNGTHWFVTQRNFYEPQIITTDNTFVSGNCDVVIGIVNNTVYDPLQPELDSFKEELDKYFKNHTSPDVDLGDISGINASVVNIQKEIDRLNEVAKNLNESLIDLQEL
GKYEQYIKWPWYIWLGFIAGLIAIVMVTIMLCCMTSCCSCLKGCCSCGSCCKFDEDDSEPVLKGVKLHYT

BA.2 S

MFVFLVLLPLVSSQCVNLITRTQSYTNSFTRGVYYPDKVFRSSVLHSTQDLFLPFFSNVTWFHAIHVSGTNGTKRFDNPVLPFNDGVYFASTEKSNIIRGWIFGTTLDSKT
QSLLIVNNATNVVIKVCEFQFCNDPFLDVYYHKNNKSWMESEFRVYSSANNCTFEYVSQPFLMDLEGKQGNFKNLREFVFKNIDGYFKIYSKHTPINLGRDLPQGFSALEPLVDLPIGINI
TRFQTLLALHRSYLTPGDSSSGWTAGAAAYYVGYLQPRTFLLKYNENGTITDAVDCALDPLSETKCTLKSFTVEKGIYQTSNFRVQPTESIVRFPNITNLCPFDEVFNATRFASVYAWNRK
RISNCVADYSVLYNFAPFFAFKCYGVSPTKLNDLCFTNVYADSFVIRGNEVSQIAPGQTGNIADYNYKLPDDFTGCVIAWNSNKLDSKVGGNYNYLYRLFRKSNLKPFERDISTEIYQAGN
KPCNGVAGFNCYFPLRSYGFRPTYGVGHQPYRVVVLSFELLHAPATVCGPKKSTNLVKNKCVNFNFNGLTGTGVLTESNKKFLPFQQFGRDIADTTDAVRDPQTLEILDITPCSFGGVSVI
TPGTNTSNQVAVLYQGVNCTEVPVAIHADQLTPTWRVYSTGSNVFQTRAGCLIGAEYVNNSYECDIPIGAGICASYQTQTKSHRRARSVASQSIIAYTMSLGAENSVAYSNNSIAIPTNFT
ISVTTEILPVSMTKTSVDCTMYICGDSTECSNLLLQYGSFCTQLKRALTGIAVEQDKNTQEVFAQVKQIYKTPPIKYFGGFNFSQILPDPSKPSKRSFIEDLLFNKVTLADAGFIKQYGDC
LGDIAARDLICAQKFNGLTVLPPLLTDEMIAQYTSALLAGTITSGWTFGAGAALQIPFAMQMAYRFNGIGVTQNVLYENQKLIANQFNSAIGKIQDSLSSTASALGKLQDVVNHNAQALNT
LVKQLSSKFGAISSVLNDILSRLDKVEAEVQIDRLITGRLQSLQTYVTQQLIRAAEIRASANLAATKMSECVLGQSKRVDFCGKGYHLMSFPQSAPHGVVFLHVTYVPAQEKNFTTAPAIC
HDGKAHFPREGVFVSNGTHWFVTQRNFYEPQIITTDNTFVSGNCDVVIGIVNNTVYDPLQPELDSFKEELDKYFKNHTSPDVDLGDISGINASVVNIQKEIDRLNEVAKNLNESLIDLQEL
GKYEQYIKWPWYIWLGFIAGLIAIVMVTIMLCCMTSCCSCLKGCCSCGSCCKFDEDDSEPVLKGVKLHYT

BA.2 S1

MFVFLVLLPLVSSQCVNLITRTQSYTNSFTRGVYYPDKVFRSSVLHSTQDLFLPFFSNVTWFHAIHVSGTNGTKRFDNPVLPFNDGVYFASTEKSNIIRGWIFGTTLDSKT
QSLLIVNNATNVVIKVCEFQFCNDPFLDVYYHKNNKSWMESEFRVYSSANNCTFEYVSQPFLMDLEGKQGNFKNLREFVFKNIDGYFKIYSKHTPINLGRDLPQGFSALEPLVDLPIGINI
TRFQTLLALHRSYLTPGDSSSGWTAGAAAYYVGYLQPRTFLLKYNENGTITDAVDCALDPLSETKCTLKSFTVEKGIYQTSNFRVQPTESIVRFPNITNLCPFDEVFNATRFASVYAWNRK
RISNCVADYSVLYNFAPFFAFKCYGVSPTKLNDLCFTNVYADSFVIRGNEVSQIAPGQTGNIADYNYKLPDDFTGCVIAWNSNKLDSKVGGNYNYLYRLFRKSNLKPFERDISTEIYQAGN
KPCNGVAGFNCYFPLRSYGFRPTYGVGHQPYRVVVLSFELLHAPATVCGPKKSTNLVKNKCVNFNFNGLTGTGVLTESNKKFLPFQQFGRDIADTTDAVRDPQTLEILDITPCSFGGVSVI
TPGTNTSNQVAVLYQGVNCTEVPVAIHADQLTPTWRVYSTGSNVFQTRAGCLIGAEYVNNSYECDIPIGAGICASYQTQTKSHRRAR

BA.2 S2

SVASQSIIAYTMSLGAENSVAYSNNSIAIPTNFTISVTTEILPVSMTKTSVDCTMYICGDSTECSNLLLQYGSFCTQLKRALTGIAVEQDKNTQEVFAQVKQIYKTPPIKY
FGGFNFSQILPDPSKPSKRSFIEDLLFNKVTLADAGFIKQYGDCLGDIAARDLICAQKFNGLTVLPPLLTDEMIAQYTSALLAGTITSGWTFGAGAALQIPFAMQMAYRFNGIGVTQNVLY
ENQKLIANQFNSAIGKIQDSLSSTASALGKLQDVVNHNAQALNTLVKQLSSKFGAISSVLNDILSRLDKVEAEVQIDRLITGRLQSLQTYVTQQLIRAAEIRASANLAATKMSECVLGQSK
RVDFCGKGYHLMSFPQSAPHGVVFLHVTYVPAQEKNFTTAPAICHDGKAHFPREGVFVSNGTHWFVTQRNFYEPQIITTDNTFVSGNCDVVIGIVNNTVYDPLQPELDSFKEELDKYFKNH
TSPDVDLGDISGINASVVNIQKEIDRLNEVAKNLNESLIDLQELGKYEQYIKWPWYIWLGFIAGLIAIVMVTIMLCCMTSCCSCLKGCCSCGSCCKFDEDDSEPVLKGVKLHYT

BA.2 S2'

SFIEDLLFNKVTLADAGFIKQYGDCLGDIAARDLICAQKFNGLTVLPPLLTDEMIAQYTSALLAGTITSGWTFGAGAALQIPFAMQMAYRFNGIGVTQNVLYENQKLIANQ
FNSAIGKIQDSLSSTASALGKLQDVVNHNAQALNTLVKQLSSKFGAISSVLNDILSRLDKVEAEVQIDRLITGRLQSLQTYVTQQLIRAAEIRASANLAATKMSECVLGQSKRVDFCGKGY
HLMSFPQSAPHGVVFLHVTYVPAQEKNFTTAPAICHDGKAHFPREGVFVSNGTHWFVTQRNFYEPQIITTDNTFVSGNCDVVIGIVNNTVYDPLQPELDSFKEELDKYFKNHTSPDVDLGD
ISGINASVVNIQKEIDRLNEVAKNLNESLIDLQELGKYEQYIKWPWYIWLGFIAGLIAIVMVTIMLCCMTSCCSCLKGCCSCGSCCKFDEDDSEPVLKGVKLHYT

BA.1 S

MFVFLVLLPLVSSQCVNLTTRTQLPPAYTNSFTRGVYYPDKVFRSSVLHSTQDLFLPFFSNVTWFHVISGTNGTKRFDNPVLPFNDGVYFASIEKSNIIRGWIFGTTLDSK
TQSLLIVNNATNVVIKVCEFQFCNDPFLDHKNNKSWMESEFRVYSSANNCTFEYVSQPFLMDLEGKQGNFKNLREFVFKNIDGYFKIYSKHTPIIVREPEDLPQGFSALEPLVDLPIGINI
TRFQTLLALHRSYLTPGDSSSGWTAGAAAYYVGYLQPRTFLLKYNENGTITDAVDCALDPLSETKCTLKSFTVEKGIYQTSNFRVQPTESIVRFPNITNLCPFDEVFNATRFASVYAWNRK
RISNCVADYSVLYNLAPFFTFKCYGVSPTKLNDLCFTNVYADSFVIRGDEVRQIAPGQTGNIADYNYKLPDDFTGCVIAWNSNKLDSKVSGNYNYLYRLFRKSNLKPFERDISTEIYQAGN
KPCNGVAGFNCYFPLRSYSFRPTYGVGHQPYRVVVLSFELLHAPATVCGPKKSTNLVKNKCVNFNFNGLKGTGVLTESNKKFLPFQQFGRDIADTTDAVRDPQTLEILDITPCSFGGVSVI
TPGTNTSNQVAVLYQGVNCTEVPVAIHADQLTPTWRVYSTGSNVFQTRAGCLIGAEYVNNSYECDIPIGAGICASYQTQTKSHRRARSVASQSIIAYTMSLGAENSVAYSNNSIAIPTNFT
ISVTTEILPVSMTKTSVDCTMYICGDSTECSNLLLQYGSFCTQLKRALTGIAVEQDKNTQEVFAQVKQIYKTPPIKYFGGFNFSQILPDPSKPSKRSFIEDLLFNKVTLADAGFIKQYGDC
LGDIAARDLICAQKFKGLTVLPPLLTDEMIAQYTSALLAGTITSGWTFGAGAALQIPFAMQMAYRFNGIGVTQNVLYENQKLIANQFNSAIGKIQDSLSSTASALGKLQDVVNHNAQALNT
LVKQLSSKFGAISSVLNDIFSRLDKVEAEVQIDRLITGRLQSLQTYVTQQLIRAAEIRASANLAATKMSECVLGQSKRVDFCGKGYHLMSFPQSAPHGVVFLHVTYVPAQEKNFTTAPAIC
HDGKAHFPREGVFVSNGTHWFVTQRNFYEPQIITTDNTFVSGNCDVVIGIVNNTVYDPLQPELDSFKEELDKYFKNHTSPDVDLGDISGINASVVNIQKEIDRLNEVAKNLNESLIDLQEL
GKYEQYIKWPWYIWLGFIAGLIAIVMVTIMLCCMTSCCSCLKGCCSCGSCCKFDEDDSEPVLKGVKLHYT

BA.1 S2

SVASQSIIAYTMSLGAENSVAYSNNSIAIPTNFTISVTTEILPVSMTKTSVDCTMYICGDSTECSNLLLQYGSFCTQLKRALTGIAVEQDKNTQEVFAQVKQIYKTPPIKY
FGGFNFSQILPDPSKPSKRSFIEDLLFNKVTLADAGFIKQYGDCLGDIAARDLICAQKFKGLTVLPPLLTDEMIAQYTSALLAGTITSGWTFGAGAALQIPFAMQMAYRFNGIGVTQNVLY
ENQKLIANQFNSAIGKIQDSLSSTASALGKLQDVVNHNAQALNTLVKQLSSKFGAISSVLNDIFSRLDKVEAEVQIDRLITGRLQSLQTYVTQQLIRAAEIRASANLAATKMSECVLGQSK
RVDFCGKGYHLMSFPQSAPHGVVFLHVTYVPAQEKNFTTAPAICHDGKAHFPREGVFVSNGTHWFVTQRNFYEPQIITTDNTFVSGNCDVVIGIVNNTVYDPLQPELDSFKEELDKYFKNH
TSPDVDLGDISGINASVVNIQKEIDRLNEVAKNLNESLIDLQELGKYEQYIKWPWYIWLGFIAGLIAIVMVTIMLCCMTSCCSCLKGCCSCGSCCKFDEDDSEPVLKGVKLHYT

BA.1 S2'

SFIEDLLFNKVTLADAGFIKQYGDCLGDIAARDLICAQKFKGLTVLPPLLTDEMIAQYTSALLAGTITSGWTFGAGAALQIPFAMQMAYRFNGIGVTQNVLYENQKLIANQ
FNSAIGKIQDSLSSTASALGKLQDVVNHNAQALNTLVKQLSSKFGAISSVLNDIFSRLDKVEAEVQIDRLITGRLQSLQTYVTQQLIRAAEIRASANLAATKMSECVLGQSKRVDFCGKGY
HLMSFPQSAPHGVVFLHVTYVPAQEKNFTTAPAICHDGKAHFPREGVFVSNGTHWFVTQRNFYEPQIITTDNTFVSGNCDVVIGIVNNTVYDPLQPELDSFKEELDKYFKNHTSPDVDLGD
ISGINASVVNIQKEIDRLNEVAKNLNESLIDLQELGKYEQYIKWPWYIWLGFIAGLIAIVMVTIMLCCMTSCCSCLKGCCSCGSCCKFDEDDSEPVLKGVKLHYT

Delta S

MFVFLVLLPLVSSQCVNLRTRTQLPPAYTNSFTRGVYYPDKVFRSSVLHSTQDLFLPFFSNVTWFHAIHVSGTNGTKRFDNPVLPFNDGVYFASTEKSNIIRGWIFGTTLD
SKTQSLLIVNNATNVVIKVCEFQFCNDPFLDVYYHKNNKSWMESEVYSSANNCTFEYVSQPFLMDLEGKQGNFKNLREFVFKNIDGYFKIYSKHTPINLVRDLPQGFSALEPLVDLPIGIN
ITRFQTLLALHRSYLTPGDSSSGWTAGAAAYYVGYLQPRTFLLKYNENGTITDAVDCALDPLSETKCTLKSFTVEKGIYQTSNFRVQPTESIVRFPNITNLCPFGEVFNATRFASVYAWNR
KRISNCVADYSVLYNSASFSTFKCYGVSPTKLNDLCFTNVYADSFVIRGDEVRQIAPGQTGKIADYNYKLPDDFTGCVIAWNSNNLDSKVGGNYNYRYRLFRKSNLKPFERDISTEIYQAG
SKPCNGVEGFNCYFPLQSYGFQPTNGVGYQPYRVVVLSFELLHAPATVCGPKKSTNLVKNKCVNFNFNGLTGTGVLTESNKKFLPFQQFGRDIADTTDAVRDPQTLEILDITPCSFGGVSV
ITPGTNTSNQVAVLYQGVNCTEVPVAIHADQLTPTWRVYSTGSNVFQTRAGCLIGAEHVNNSYECDIPIGAGICASYQTQTNSRRRARSVASQSIIAYTMSLGAENSVAYSNNSIAIPTNF
TISVTTEILPVSMTKTSVDCTMYICGDSTECSNLLLQYGSFCTQLNRALTGIAVEQDKNTQEVFAQVKQIYKTPPIKDFGGFNFSQILPDPSKPSKRSFIEDLLFNKVTLADAGFIKQYGD
CLGDIAARDLICAQKFNGLTVLPPLLTDEMIAQYTSALLAGTITSGWTFGAGAALQIPFAMQMAYRFNGIGVTQNVLYENQKLIANQFNSAIGKIQDSLSSTASALGKLQNVVNQNAQALN
TLVKQLSSNFGAISSVLNDILSRLDKVEAEVQIDRLITGRLQSLQTYVTQQLIRAAEIRASANLAATKMSECVLGQSKRVDFCGKGYHLMSFPQSAPHGVVFLHVTYVPAQEKNFTTAPAI
CHDGKAHFPREGVFVSNGTHWFVTQRNFYEPQIITTDNTFVSGNCDVVIGIVNNTVYDPLQPELDSFKEELDKYFKNHTSPDVDLGDISGINASVVNIQKEIDRLNEVAKNLNESLIDLQE
LGKYEQYIKWPWYIWLGFIAGLIAIVMVTIMLCCMTSCCSCLKGCCSCGSCCKFDEDDSEPVLKGVKLHYT

Delta S1

MFVFLVLLPLVSSQCVNLRTRTQLPPAYTNSFTRGVYYPDKVFRSSVLHSTQDLFLPFFSNVTWFHAIHVSGTNGTKRFDNPVLPFNDGVYFASTEKSNIIRGWIFGTTLD
SKTQSLLIVNNATNVVIKVCEFQFCNDPFLDVYYHKNNKSWMESEVYSSANNCTFEYVSQPFLMDLEGKQGNFKNLREFVFKNIDGYFKIYSKHTPINLVRDLPQGFSALEPLVDLPIGIN
ITRFQTLLALHRSYLTPGDSSSGWTAGAAAYYVGYLQPRTFLLKYNENGTITDAVDCALDPLSETKCTLKSFTVEKGIYQTSNFRVQPTESIVRFPNITNLCPFGEVFNATRFASVYAWNR
KRISNCVADYSVLYNSASFSTFKCYGVSPTKLNDLCFTNVYADSFVIRGDEVRQIAPGQTGKIADYNYKLPDDFTGCVIAWNSNNLDSKVGGNYNYRYRLFRKSNLKPFERDISTEIYQAG
SKPCNGVEGFNCYFPLQSYGFQPTNGVGYQPYRVVVLSFELLHAPATVCGPKKSTNLVKNKCVNFNFNGLTGTGVLTESNKKFLPFQQFGRDIADTTDAVRDPQTLEILDITPCSFGGVSV
ITPGTNTSNQVAVLYQGVNCTEVPVAIHADQLTPTWRVYSTGSNVFQTRAGCLIGAEHVNNSYECDIPIGAGICASYQTQTNSRRRAR

Alpha S

MFVFLVLLPLVSSQCVNLTTRTQLPPAYTNSFTRGVYYPDKVFRSSVLHSTQDLFLPFFSNVTWFHAISGTNGTKRFDNPVLPFNDGVYFASTEKSNIIRGWIFGTTLDSK
TQSLLIVNNATNVVIKVCEFQFCNDPFLGVYHKNNKSWMESEFRVYSSANNCTFEYVSQPFLMDLEGKQGNFKNLREFVFKNIDGYFKIYSKHTPINLVRDLPQGFSALEPLVDLPIGINI
TRFQTLLALHRSYLTPGDSSSGWTAGAAAYYVGYLQPRTFLLKYNENGTITDAVDCALDPLSETKCTLKSFTVEKGIYQTSNFRVQPTESIVRFPNITNLCPFGEVFNATRFASVYAWNRK
RISNCVADYSVLYNSASFSTFKCYGVSPTKLNDLCFTNVYADSFVIRGDEVRQIAPGQTGKIADYNYKLPDDFTGCVIAWNSNNLDSKVGGNYNYLYRLFRKSNLKPFERDISTEIYQAGS
TPCNGVEGFNCYFPLQSYGFQPTYGVGYQPYRVVVLSFELLHAPATVCGPKKSTNLVKNKCVNFNFNGLTGTGVLTESNKKFLPFQQFGRDIDDTTDAVRDPQTLEILDITPCSFGGVSVI
TPGTNTSNQVAVLYQGVNCTEVPVAIHADQLTPTWRVYSTGSNVFQTRAGCLIGAEHVNNSYECDIPIGAGICASYQTQTNSHRRARSVASQSIIAYTMSLGAENSVAYSNNSIAIPINFT
ISVTTEILPVSMTKTSVDCTMYICGDSTECSNLLLQYGSFCTQLNRALTGIAVEQDKNTQEVFAQVKQIYKTPPIKDFGGFNFSQILPDPSKPSKRSFIEDLLFNKVTLADAGFIKQYGDC
LGDIAARDLICAQKFNGLTVLPPLLTDEMIAQYTSALLAGTITSGWTFGAGAALQIPFAMQMAYRFNGIGVTQNVLYENQKLIANQFNSAIGKIQDSLSSTASALGKLQDVVNQNAQALNT
LVKQLSSNFGAISSVLNDILARLDKVEAEVQIDRLITGRLQSLQTYVTQQLIRAAEIRASANLAATKMSECVLGQSKRVDFCGKGYHLMSFPQSAPHGVVFLHVTYVPAQEKNFTTAPAIC
HDGKAHFPREGVFVSNGTHWFVTQRNFYEPQIITTHNTFVSGNCDVVIGIVNNTVYDPLQPELDSFKEELDKYFKNHTSPDVDLGDISGINASVVNIQKEIDRLNEVAKNLNESLIDLQEL
GKYEQYIKWPWYIWLGFIAGLIAIVMVTIMLCCMTSCCSCLKGCCSCGSCCKFDEDDSEPVLKGVKLHYT

Alpha S1

MFVFLVLLPLVSSQCVNLTTRTQLPPAYTNSFTRGVYYPDKVFRSSVLHSTQDLFLPFFSNVTWFHAISGTNGTKRFDNPVLPFNDGVYFASTEKSNIIRGWIFGTTLDSK
TQSLLIVNNATNVVIKVCEFQFCNDPFLGVYHKNNKSWMESEFRVYSSANNCTFEYVSQPFLMDLEGKQGNFKNLREFVFKNIDGYFKIYSKHTPINLVRDLPQGFSALEPLVDLPIGINI
TRFQTLLALHRSYLTPGDSSSGWTAGAAAYYVGYLQPRTFLLKYNENGTITDAVDCALDPLSETKCTLKSFTVEKGIYQTSNFRVQPTESIVRFPNITNLCPFGEVFNATRFASVYAWNRK
RISNCVADYSVLYNSASFSTFKCYGVSPTKLNDLCFTNVYADSFVIRGDEVRQIAPGQTGKIADYNYKLPDDFTGCVIAWNSNNLDSKVGGNYNYLYRLFRKSNLKPFERDISTEIYQAGS
TPCNGVEGFNCYFPLQSYGFQPTYGVGYQPYRVVVLSFELLHAPATVCGPKKSTNLVKNKCVNFNFNGLTGTGVLTESNKKFLPFQQFGRDIDDTTDAVRDPQTLEILDITPCSFGGVSVI
TPGTNTSNQVAVLYQGVNCTEVPVAIHADQLTPTWRVYSTGSNVFQTRAGCLIGAEHVNNSYECDIPIGAGICASYQTQTNSHRRAR

SARS-CoV-2 S

MFVFLVLLPLVSSQCVNLTTRTQLPPAYTNSFTRGVYYPDKVFRSSVLHSTQDLFLPFFSNVTWFHAIHVSGTNGTKRFDNPVLPFNDGVYFASTEKSNIIRGWIFGTTLD
SKTQSLLIVNNATNVVIKVCEFQFCNDPFLGVYYHKNNKSWMESEFRVYSSANNCTFEYVSQPFLMDLEGKQGNFKNLREFVFKNIDGYFKIYSKHTPINLVRDLPQGFSALEPLVDLPIG
INITRFQTLLALHRSYLTPGDSSSGWTAGAAAYYVGYLQPRTFLLKYNENGTITDAVDCALDPLSETKCTLKSFTVEKGIYQTSNFRVQPTESIVRFPNITNLCPFGEVFNATRFASVYAW
NRKRISNCVADYSVLYNSASFSTFKCYGVSPTKLNDLCFTNVYADSFVIRGDEVRQIAPGQTGKIADYNYKLPDDFTGCVIAWNSNNLDSKVGGNYNYLYRLFRKSNLKPFERDISTEIYQ
AGSTPCNGVEGFNCYFPLQSYGFQPTNGVGYQPYRVVVLSFELLHAPATVCGPKKSTNLVKNKCVNFNFNGLTGTGVLTESNKKFLPFQQFGRDIADTTDAVRDPQTLEILDITPCSFGGV
SVITPGTNTSNQVAVLYQDVNCTEVPVAIHADQLTPTWRVYSTGSNVFQTRAGCLIGAEHVNNSYECDIPIGAGICASYQTQTNSPRRARSVASQSIIAYTMSLGAENSVAYSNNSIAIPT
NFTISVTTEILPVSMTKTSVDCTMYICGDSTECSNLLLQYGSFCTQLNRALTGIAVEQDKNTQEVFAQVKQIYKTPPIKDFGGFNFSQILPDPSKPSKRSFIEDLLFNKVTLADAGFIKQY
GDCLGDIAARDLICAQKFNGLTVLPPLLTDEMIAQYTSALLAGTITSGWTFGAGAALQIPFAMQMAYRFNGIGVTQNVLYENQKLIANQFNSAIGKIQDSLSSTASALGKLQDVVNQNAQA
LNTLVKQLSSNFGAISSVLNDILSRLDKVEAEVQIDRLITGRLQSLQTYVTQQLIRAAEIRASANLAATKMSECVLGQSKRVDFCGKGYHLMSFPQSAPHGVVFLHVTYVPAQEKNFTTAP
AICHDGKAHFPREGVFVSNGTHWFVTQRNFYEPQIITTDNTFVSGNCDVVIGIVNNTVYDPLQPELDSFKEELDKYFKNHTSPDVDLGDISGINASVVNIQKEIDRLNEVAKNLNESLIDL
QELGKYEQYIKWPWYIWLGFIAGLIAIVMVTIMLCCMTSCCSCLKGCCSCGSCCKFDEDDSEPVLKGVKLHYT

SARS-CoV-2 S1

MFVFLVLLPLVSSQCVNLTTRTQLPPAYTNSFTRGVYYPDKVFRSSVLHSTQDLFLPFFSNVTWFHAIHVSGTNGTKRFDNPVLPFNDGVYFASTEKSNIIRGWIFGTTLD
SKTQSLLIVNNATNVVIKVCEFQFCNDPFLGVYYHKNNKSWMESEFRVYSSANNCTFEYVSQPFLMDLEGKQGNFKNLREFVFKNIDGYFKIYSKHTPINLVRDLPQGFSALEPLVDLPIG
INITRFQTLLALHRSYLTPGDSSSGWTAGAAAYYVGYLQPRTFLLKYNENGTITDAVDCALDPLSETKCTLKSFTVEKGIYQTSNFRVQPTESIVRFPNITNLCPFGEVFNATRFASVYAW
NRKRISNCVADYSVLYNSASFSTFKCYGVSPTKLNDLCFTNVYADSFVIRGDEVRQIAPGQTGKIADYNYKLPDDFTGCVIAWNSNNLDSKVGGNYNYLYRLFRKSNLKPFERDISTEIYQ
AGSTPCNGVEGFNCYFPLQSYGFQPTNGVGYQPYRVVVLSFELLHAPATVCGPKKSTNLVKNKCVNFNFNGLTGTGVLTESNKKFLPFQQFGRDIADTTDAVRDPQTLEILDITPCSFGGV
SVITPGTNTSNQVAVLYQDVNCTEVPVAIHADQLTPTWRVYSTGSNVFQTRAGCLIGAEHVNNSYECDIPIGAGICASYQTQTNSPRRAR

SARS-CoV-2 S2

SVASQSIIAYTMSLGAENSVAYSNNSIAIPTNFTISVTTEILPVSMTKTSVDCTMYICGDSTECSNLLLQYGSFCTQLNRALTGIAVEQDKNTQEVFAQVKQIYKTPPIKD
FGGFNFSQILPDPSKPSKRSFIEDLLFNKVTLADAGFIKQYGDCLGDIAARDLICAQKFNGLTVLPPLLTDEMIAQYTSALLAGTITSGWTFGAGAALQIPFAMQMAYRFNGIGVTQNVLY
ENQKLIANQFNSAIGKIQDSLSSTASALGKLQDVVNQNAQALNTLVKQLSSNFGAISSVLNDILSRLDKVEAEVQIDRLITGRLQSLQTYVTQQLIRAAEIRASANLAATKMSECVLGQSK
RVDFCGKGYHLMSFPQSAPHGVVFLHVTYVPAQEKNFTTAPAICHDGKAHFPREGVFVSNGTHWFVTQRNFYEPQIITTDNTFVSGNCDVVIGIVNNTVYDPLQPELDSFKEELDKYFKNH
TSPDVDLGDISGINASVVNIQKEIDRLNEVAKNLNESLIDLQELGKYEQYIKWPWYIWLGFIAGLIAIVMVTIMLCCMTSCCSCLKGCCSCGSCCKFDEDDSEPVLKGVKLHYT

SARS-CoV-2 S2’

SFIEDLLFNKVTLADAGFIKQYGDCLGDIAARDLICAQKFNGLTVLPPLLTDEMIAQYTSALLAGTITSGWTFGAGAALQIPFAMQMAYRFNGIGVTQNVLYENQKLIANQ
FNSAIGKIQDSLSSTASALGKLQDVVNQNAQALNTLVKQLSSNFGAISSVLNDILSRLDKVEAEVQIDRLITGRLQSLQTYVTQQLIRAAEIRASANLAATKMSECVLGQSKRVDFCGKGY
HLMSFPQSAPHGVVFLHVTYVPAQEKNFTTAPAICHDGKAHFPREGVFVSNGTHWFVTQRNFYEPQIITTDNTFVSGNCDVVIGIVNNTVYDPLQPELDSFKEELDKYFKNHTSPDVDLGD
ISGINASVVNIQKEIDRLNEVAKNLNESLIDLQELGKYEQYIKWPWYIWLGFIAGLIAIVMVTIMLCCMTSCCSCLKGCCSCGSCCKFDEDDSEPVLKGVKLHYT

BA.4 N

MSDNGPQNQRNAPRITFGGPSDSTGSNQNGERSGARSKQRRPQGLPNNTASWFTALTQHGKEDLKFPRGQGVPINTNSSPDDQIGYYRRATRRIRGGDGKMKDLSPRWYFY
YLGTGPEAGLPYGANKDGIIWVATEGALNTPKDHIGTRNSANNAAIVLQLPQGTTLPKGFYAEGSRGGSQASSRSSSRSRNSSRNSTPGSSKRTSPARMAGNGGDAALALLLLDRLNQLES
KMSGKGQQQQGQTVTKKSAAEASKKPRQKRTATKAYNVTQAFGRRGPEQTQGNFGDQELIRQGTDYKHWPQIAQFAPSASAFFGMSRIGMEVTPSGTWLTYTGAIKLDDKDPNFKDQVILL
NKHIDAYKTFPPTEPKKDKKKKADETQALPQRQKKQQTVTLLPAADLDDFSKQLQQSMSRADSTQA

SARS-CoV-2 N

MSDNGPQNQRNAPRITFGGPSDSTGSNQNGERSGARSKQRRPQGLPNNTASWFTALTQHGKEDLKFPRGQGVPINTNSSPDDQIGYYRRATRRIRGGDGKMKDLSPRWYFY
YLGTGPEAGLPYGANKDGIIWVATEGALNTPKDHIGTRNPANNAAIVLQLPQGTTLPKGFYAEGSRGGSQASSRSSSRSRNSSRNSTPGSSRGTSPARMAGNGGDAALALLLLDRLNQLES
KMSGKGQQQQGQTVTKKSAAEASKKPRQKRTATKAYNVTQAFGRRGPEQTQGNFGDQELIRQGTDYKHWPQIAQFAPSASAFFGMSRIGMEVTPSGTWLTYTGAIKLDDKDPNFKDQVILL
NKHIDAYKTFPPTEPKKDKKKKADETQALPQRQKKQQTVTLLPAADLDDFSKQLQQSMSSADSTQA

BA.5 M

MANSNGTITVEELKKLLEEWNLVIGFLFLTWICLLQFAYANRNRFLYIIKLIFLWLLWPVTLTCFVLAAVYRINWITGGIAIAMACLVGLMWLSYFIASFRLFARTRSMWS
FNPETNILLNVPLHGTILTRPLLESELVIGAVILRGHLRIAGHHLGRCDIKDLPKEITVATSRTLSYYKLGASQRVAGDSGFAAYSRYRIGNYKLNTDHSSSSDNIALLVQ

SARS-CoV-2 M

MADSNGTITVEELKKLLEQWNLVIGFLFLTWICLLQFAYANRNRFLYIIKLIFLWLLWPVTLACFVLAAVYRINWITGGIAIAMACLVGLMWLSYFIASFRLFARTRSMWS
FNPETNILLNVPLHGTILTRPLLESELVIGAVILRGHLRIAGHHLGRCDIKDLPKEITVATSRTLSYYKLGASQRVAGDSGFAAYSRYRIGNYKLNTDHSSSSDNIALLVQ

HIV-1

MRVKGIRKNYQHLWRGGTLLLGIIVICSAVEKLWVTVYYGVPVWKEATTTLFCASDAKAYDTEVHNVWATHACVPTDPNPQEVVLGNVTEKFNMWKNNMVEQMQEDIISLW
DQSLKPCVKLTPLCVTLNCKDVNATNTTNGSEGTMERGEIKNCSFNITTSIRDEVQKEYALFYKLDVVPIDNNNTSYRLISCDTSVITQACPKISFEPIPIHYCAPAGFAILKCNDKTFNG
KGPCKNVSTVQCTHGIRPVVSTQLLLNGSLAEEEVVIRSDNFTNNAKTIIVQLKESVEINCTRPNNNTRKSIHIGPGRAFYTTGEIIGDIRQAHCNISRAKWNDTLKQIVIKLREQFENKT
IVFNHSSGGDPEIVMHSFNCGGEFFYCNSTQLFNSTWNNNTEGSNNTEGNTITLPCRIKQIINMWQEVGKAMYAPPIRGQIRCSSNITGLLLTRDGGINENGTEIFRPGGGDMKDNWRSEL
YKYKVVKIEPLGVAPTKAKRRVVQREKRAVGIGAVFLGFLGAAGSTMGAASMTLTVQARLLLSGIVQQQNNLLRAIEAQQRMLQLTVWGIKQLQARVLAVERYLGDQQLLGIWGCSGKLIC
TTAVPWNASWSNKSLDRIWNNMTWMEWEREIDNYTSEIYTLIEESQNQQEKNEQELLELDKWASLWNWFDITKWLWYIKIFIMIVGGLIGLRIVFTVLSIVNRVRQGYSPLSFQTLLPAPR
GPDRPEGIEEEGGERDRDRSGRLVNGFLALIWVDLRSLCLFSYHRLRDLLLTVTRIVELLGRRGWEVLKYWWNLLQYWSQELKNSAVSLLNATAIAVAEGTDRIIEALQRTYRAILHIPTR
IRQGLERALL

HIV-1 furin-cleaved

MRVKGIRKNYQHLWRGGTLLLGIIVICSAVEKLWVTVYYGVPVWKEATTTLFCASDAKAYDTEVHNVWATHACVPTDPNPQEVVLGNVTEKFNMWKNNMVEQMQEDIISLW
DQSLKPCVKLTPLCVTLNCKDVNATNTTNGSEGTMERGEIKNCSFNITTSIRDEVQKEYALFYKLDVVPIDNNNTSYRLISCDTSVITQACPKISFEPIPIHYCAPAGFAILKCNDKTFNG
KGPCKNVSTVQCTHGIRPVVSTQLLLNGSLAEEEVVIRSDNFTNNAKTIIVQLKESVEINCTRPNNNTRKSIHIGPGRAFYTTGEIIGDIRQAHCNISRAKWNDTLKQIVIKLREQFENKT
IVFNHSSGGDPEIVMHSFNCGGEFFYCNSTQLFNSTWNNNTEGSNNTEGNTITLPCRIKQIINMWQEVGKAMYAPPIRGQIRCSSNITGLLLTRDGGINENGTEIFRPGGGDMKDNWRSEL
YKYKVVKIEPLGVAPTKAKRRVVQREKR

Influenza A (avian)

MEEIVLLFAIVSLARSDQICIGYHANNSTKQVDTIMEKNVTVTHAQDILEKTHNGKLCSLNGVKPLILRDCSVAGWLLGNPMCDEFLNVPEWSYIVEKDNPVNGLCYPGDF
NDYEELKHLLSCTKHFEKIRIIPRDSWPNHEASLGVSSACPYNGRSSFFRNVVWLIKKDNAYPTIKRSYNNTNKEDLLILWGIHHPNDAAEQTKLYQNPTTYVSVGTSTLNQRSIPKIATR
PKLNGQSGRMEFFWTILKPSDTINFESNGNFIAPEYAYKIVKKGDSAIMKSGLEYGNCNTKCQTPIGAINSSMPFHNIHPLTIGECPKYVKSDRLVLATGLRNTPQRKRKKRGLFGAIAGF
IEGGWQGMVDGWYGYHHSNEQGSGYAADKESTQKAIDGITNKVNSIIDKMNTQFEAVGKEFNNLERRIENLNKILEDGFLDVWTYNAELLVLMENERTLDFHEANVKSLYDKVRLQLKDNA
RELGNGCFEFYHKCDNECMESIRNGTYNYPQYSEEARLNREEISGIKLESMGIYQILSIYSTVASSLALAIMIAGLSFWMCSNGSLQCRICI

Influenza A (avian) furin-cleaved

MEEIVLLFAIVSLARSDQICIGYHANNSTKQVDTIMEKNVTVTHAQDILEKTHNGKLCSLNGVKPLILRDCSVAGWLLGNPMCDEFLNVPEWSYIVEKDNPVNGLCYPGDF
NDYEELKHLLSCTKHFEKIRIIPRDSWPNHEASLGVSSACPYNGRSSFFRNVVWLIKKDNAYPTIKRSYNNTNKEDLLILWGIHHPNDAAEQTKLYQNPTTYVSVGTSTLNQRSIPKIATR
PKLNGQSGRMEFFWTILKPSDTINFESNGNFIAPEYAYKIVKKGDSAIMKSGLEYGNCNTKCQTPIGAINSSMPFHNIHPLTIGECPKYVKSDRLVLATGLRNTPQRKRKKR

Measles

MGLKVNVSAIFMAVLLTLQTPTGQIHWGNLSKIGVVGIGSASYKVMTRSSHQSLVIKLMPNITLLNNCTRVEIAEYRRLLRTVLEPIRDALNAMTQNIRPVQSVASSRRHK
RFAGVVLAGAALGVATAAQITAGIALHQSMLNSQAIDNLRASLETTNQAIEAIRQAGQEMILAVQGVQDYINNELIPSMNQLSCDLIGQKLGLKLLRYYTEILSLFGPSLRDPISAEISIQ
ALSYALGGDINKVLEKLGYSGGDLLGILESRGIKARITHVDTESYFIVLSIAYPTLSEIKGVIVHRLEGVSYNIGSQEWYTTVPKYVATQGYLISNFDESSCTFMPEGTVCSQNALYPMSP
LLQECLRGSTKSCARTLVSGSFGNRFILSQGNLIANCASILCKCYTTGTIINQDPDKILTYIAADHCPVVEVNGVTIQVGSRRYPDAVYLHRIDLGPPISLERLDVGTNLGNAIAKLEDAK
ELLESSDQILRSMKGLSSTSIVYILIAVCLGGLIGIPALICCCRGRCNKKGEQVGMSRPGLKPDLTGTSKSYVRSL

Measles furin-cleaved

MGLKVNVSAIFMAVLLTLQTPTGQIHWGNLSKIGVVGIGSASYKVMTRSSHQSLVIKLMPNITLLNNCTRVEIAEYRRLLRTVLEPIRDALNAMTQNIRPVQSVASSRRHK
R

EBV

MTRRRVLSVVVLLAALACRLGAQTPEQPAPPATTVQPTATRQQTSFPFRVCELSSHGDLFRFSSDIQCPSFGTRENHTEGLLMVFKDNIIPYSFKVRSYTKIVTNILIYNG
WYADSVTNRHEEKFSVDSYETDQMDTIYQCYNAVKMTKDGLTRVYVDRDGVNITVNLKPTGGLANGVRRYASQTELYDAPGWLIWTYRTRTTVNCLITDMMAKSNSPFDFFVTTTGQTVEM
SPFYDGKNKETFHERADSFHVRTNYKIVDYDNRGTNPQGERRAFLDKGTYTLSWKLENRTAYCPLQHWQTFDSTIATETGKSIHFVTDEGTSSFVTNTTVGIELPDAFKCIEEQVNKTMHE
KYEAVQDRYTKGQEAITYFITSGGLLLAWLPLTPRSLATVKNLTELTTPTSSPPSSPSPPAPSAARGSTPAAVLRRRRRDAGNATTPVPPTAPGKSLGTLNNPATVQIQFAYDSLRRQINR
MLGDLARAWCLEQKRQNMVLRELTKINPTTVMSSIYGKAVAAKRLGDVISVSQCVPVNQATVTLRKSMRVPGSETMCYSRPLVSFSFINDTKTYEGQLGTDNEIFLTKKMTEVCQATSQYY
FQSGNEIHVYNDYHHFKTIELDGIATLQTFISLNTSLIENIDFASLELYSRDEQRASNVFDLEGIFREYNFQAQNIAGLRKDLDNAVSNGRNQFVDGLGELMDSLGSVGQSITNLVSTVGG
LFSSLVSGFISFFKNPFGGMLILVLVAGVVILVISLTRRTRQMSQQPVQMLYPGIDELAQQHASGEGPGINPISKTELQAIMLALHEQNQEQKRAAQRAAGPSVASRALQAARDRFPGLRR
RRYHDPETAAALLGEAETEF

EBV furin-cleaved

MTRRRVLSVVVLLAALACRLGAQTPEQPAPPATTVQPTATRQQTSFPFRVCELSSHGDLFRFSSDIQCPSFGTRENHTEGLLMVFKDNIIPYSFKVRSYTKIVTNILIYNG
WYADSVTNRHEEKFSVDSYETDQMDTIYQCYNAVKMTKDGLTRVYVDRDGVNITVNLKPTGGLANGVRRYASQTELYDAPGWLIWTYRTRTTVNCLITDMMAKSNSPFDFFVTTTGQTVEM
SPFYDGKNKETFHERADSFHVRTNYKIVDYDNRGTNPQGERRAFLDKGTYTLSWKLENRTAYCPLQHWQTFDSTIATETGKSIHFVTDEGTSSFVTNTTVGIELPDAFKCIEEQVNKTMHE
KYEAVQDRYTKGQEAITYFITSGGLLLAWLPLTPRSLATVKNLTELTTPTSSPPSSPSPPAPSAARGSTPAAVLRRRRR

HTLV-1

MGKFLATLILFFQFCPLILGDYSPSCCTLTIGVSSYHSKPCNPAQPVCSWTLDLLALSADQALQPPCPNLVSYSSYHATYSLYLFPHWIKKPNRNGGGYYSASYSDPCSLK
CPYLGCQSWTCPYTGAVSSPYWKFQQDVNFTQEVSRLNINLHFSKCGFPFSLLVDAPGYDPIWFLNTEPSQLPPTAPPLLPHSNLDHILEPSIPWKSKLLTLVQLTLQSTNYTCIVCIDRA
SLSTWHVLYSPNVSVPSSSSTPLLYPSLALPAPHLTLPFNWTHCFDPQIQAIVSSPCHNSLILPPFSLSPVPTLGSRSRRAVPVAVWLVSALAMGAGVAGGITGSMSLASGKSLLHEVDKD
ISQLTQAIVKNHKNLLKIAQYAAQNRRGLDLLFWEQGGLCKALQEQCCFLNITNSHVSILQERPPLENRVLTGWGLNWDLGLSQWAREALQTGITLVALLLLVILAGPCILRQLRHLPSRV
RYPHYSLINPESSL

HTLV-1 furin-cleaved

MGKFLATLILFFQFCPLILGDYSPSCCTLTIGVSSYHSKPCNPAQPVCSWTLDLLALSADQALQPPCPNLVSYSSYHATYSLYLFPHWIKKPNRNGGGYYSASYSDPCSLK
CPYLGCQSWTCPYTGAVSSPYWKFQQDVNFTQEVSRLNINLHFSKCGFPFSLLVDAPGYDPIWFLNTEPSQLPPTAPPLLPHSNLDHILEPSIPWKSKLLTLVQLTLQSTNYTCIVCIDRA
SLSTWHVLYSPNVSVPSSSSTPLLYPSLALPAPHLTLPFNWTHCFDPQIQAIVSSPCHNSLILPPFSLSPVPTLGSRSRR

Table S2. Supersecondary structure code (SSSC) data of protein data bank (PDB) structures.

6eqx_D RRRVRX THSSTD

7xnq_A

TNSFTRGVYYPDKVFRSSVLHSTQDLFLPFFSNVTWFHAISGTNGTKRFDNPVLPFNDGVYFASTEKSNIIRGWIFGTTLDSKTQSLLIVNNATNVVIKVCEFQFCNDPFL
DVYYHKNNKSWMESEFRVYSSANNCTFEYVSQPFLMDLEGKQGNFKNLREFVFKNIDGYFKIYSKHTPINLGRDLPQGFSALEPLVDLPIGINITRFQTLLALHRSYLTPGDSSSGWTAGA
AAYYVGYLQPRTFLLKYNENGTITDAVDCALDPLSETKCTLKSFTVEKGIYQTSNFRVQPTESIVRFPNITNLCPFDEVFNATRFASVYAWNRKRISNCVADYSVLYNFAPFFAFKCYGVS
PTKLNDLCFTNVYADSFVIRGNEVSQIAPGQTGNIADYNYKLPDDFTGCVIAWNSNKLDSKVGGNYNYRYRLFRKSNLKPFERDISTEIYQAGNKPCNGVAGVNCYFPLQSYGFRPTYGVG
HQPYRVVVLSFELLHAPATVCGPKKSTNLVKNKCVNFNFNGLTGTGVLTESNKKFLPFQQFGRDIADTTDAVRDPQTLEILDITPCSFGGVSVITPGTNTSNQVAVLYQGVNCTEVPVAIH
ADQLTPTWRVYSTGSNVFQTRAGCLIGAEYVNSSYECDIPIGAGICASYQTQXSQSIIAYTMSLGAENSVAYSNNSIAIPTNFTISVTTEILPVSMTKTSVDCTMYICGDSTECSNLLLQY
GSFCTQLKRALTGIAVEQDKNTQEVFAQVKQIYKTPPIKYFGGFNFSQILPDPSKPSKRSPIEDLLFNKVTLXLICAQKFNGLTVLPPLLTDEMIAQYTSALLAGTITSGWTFGAGPALQI
PFPMQMAYRFNGIGVTQNVLYENQKLIANQFNSAIGKIQDSLSSTPSALGKLQDVVNHNAQALNTLVKQLSSKFGAISSVLNDILSRLDPPEAEVQIDRLITGRLQSLQTYVTQQLIRAAE
IRASANLAATKMSECVLGQSKRVDFCGKGYHLMSFPQSAPHGVVFLHVTYVPAQEKNFTTAPAICHDGKAHFPREGVFVSNGTHWFVTQRNFYEPQIITTDNTFVSGNCDVVIGIVNNTVY
DPLQPELDSFKEX

TSSHHSTSSSHSTSSSSTSTSSSHSSSSSSTSSSSHTSSHTHTHTTSHSSSSSSHTTTTSSSSSSSHHTHSHSSSSTHHTSHHSSSTSSSSSHTSSSSSHSSSHSSSSSSS
SSHSHTHHTHSSSTSHTHSSSSHSSSSSTSSSSHSSSSSSSTTTSSSSSSSSSSSSTTSSSSSSSSSTSSSHSSTSSTSSSSSSHSSSTSSSSSHSSSSSSSHTSSSTSSSSTSSTTSSTS
SHSSSSSSSSSTSSSSSSHHTSSHSSSSHHHSHHHHHHHHHTSHSSSSTSSSHSSSSSSSHSSSSSSSHSTSSSHHHHHHHSHSHSSTHTSSSSSSSSSSTSHHHHHHHSSSHHTSSTSSS
THTHHHSSSHSSTSSSSSSSHHHHHHHSTHTSSHHHHHHSSSSHTSSTSSSSSSSHHHHSSSHHTSHHHSSSHSSHSSSSSTSSSSTTSSSSHSTSSHHSSSHSTSSSSSSSHTSSHTSSHH
TSSSSSSSSSSSSSSHSSSHSTTHSSSSSSSHHSSSTSTSTTSSTSTSSSSSHSSSSHHSHSSSSTSHSSHSSSSHSTTSSSTSSSSSSSSSSSSSSHTHHHSHSSSSSSSTSTHHHHHHTSS
STHSHHTTHHTHHTHSSSSSHHTSSSTSSSSHSSSSSHSSHTTTSSSSSSSTDTSSSSTSSSSHTSSSSSSSSHHSSSSSHSSSSSSSSSSSSHSSSSSSSSHHHHHSTTSHHHHHHHHHH
TTHHHHHHHHHHHHHHHHHHHHHHHHTHSHSSSSSSHSTSTTTSSHHHHSSSHHSHSTSSHHHHHHHHHSSTDTHHHHHHHTSSSSSSHSSHHHHHHHHHHHHHHHHHHTHHHHHTSSSSS
SHHHHHHHHHHHHTSSHHHHHHTHHHHHHHHHHHHHHHHHHHHHSHHHTHHHHHHHHHHHHHHHHHHHHHHHSHTTSHSHSHHHHHHHSSHHHHHHHHHHHHHHHHHHHHHHHHHHHHHHHHH
HHHHHHHHHHHHHHHHHSSSHSSTHTTSTSSHSSSSSSSSTTSSSTSSSSSSSSTSSSSSSSHSSSHTTSSSSSHSTSSSSHTHSSSSSSHHSSTSSSSSHHHSSSSTSHHHSSTSSSSSSS
SHHHHHHHHHHTD

7xnq_B

TNSFTRGVYYPDKVFRSSVLHSTQDLFLPFFSNVTWFHAISGTNGTKRFDNPVLPFNDGVYFASTEKSNIIRGWIFGTTLDSKTQSLLIVNNATNVVIKVCEFQFCNDPFL
DVYYHKNNKSWMESEFRVYSSANNCTFEYVSQPFLMDLEGKQGNFKNLREFVFKNIDGYFKIYSKHTPINLGRDLPQGFSALEPLVDLPIGINITRFQTLLALHRSYLTPGDSSSGWTAGA
AAYYVGYLQPRTFLLKYNENGTITDAVDCALDPLSETKCTLKSFTVEKGIYQTSNFRVQPTESIVRFPNITNLCPFDEVFNATRFASVYAWNRKRISNCVADYSVLYNFAPFFAFKCYGVS
PTKLNDLCFTNVYADSFVIRGNEVSQIAPGQTGNIADYNYKLPDDFTGCVIAWNSNKLDSKVGGNYNYRYRLFRKSNLKPFERDISTEIYQAGNKPCNGVAGVNCYFPLQSYGFRPTYGVG
HQPYRVVVLSFELLHAPATVCGPKKSTNLVKNKCVNFNFNGLTGTGVLTESNKKFLPFQQFGRDIADTTDAVRDPQTLEILDITPCSFGGVSVITPGTNTSNQVAVLYQGVNCTEVPVAIH
ADQLTPTWRVYSTGSNVFQTRAGCLIGAEYVNSSYECDIPIGAGICASYQTQXSQSIIAYTMSLGAENSVAYSNNSIAIPTNFTISVTTEILPVSMTKTSVDCTMYICGDSTECSNLLLQY
GSFCTQLKRALTGIAVEQDKNTQEVFAQVKQIYKTPPIKYFGGFNFSQILPDPSKPSKRSPIEDLLFNKVTLXLICAQKFNGLTVLPPLLTDEMIAQYTSALLAGTITSGWTFGAGPALQI
PFPMQMAYRFNGIGVTQNVLYENQKLIANQFNSAIGKIQDSLSSTPSALGKLQDVVNHNAQALNTLVKQLSSKFGAISSVLNDILSRLDPPEAEVQIDRLITGRLQSLQTYVTQQLIRAAE
IRASANLAATKMSECVLGQSKRVDFCGKGYHLMSFPQSAPHGVVFLHVTYVPAQEKNFTTAPAICHDGKAHFPREGVFVSNGTHWFVTQRNFYEPQIITTDNTFVSGNCDVVIGIVNNTVY
DPLQPELDSFKEX

TSSHHSTSSSHSTSSSSTSTSSSHSSSSSSTSSSSHTSSHTHTSTHSTSTSSSSHTTTTSSSSSSSHHTHSHSSSSTHHTSHHSSSTSSSSSHTSSSSSHSSSHSSSSSSS
SSHTHTHHTHSSSTSHTHSSSSHSSSSSTSSSSHSSSSSSSTTTSSSSSSSSSSSSTTSSSSSSSSSTSSSHSSTSSTSSSSSSHSSSTSSSSSHSSSSSSSTHSSTTSSSSTSSTTSSTS
SHSSSSSSSSSTSSSSSSHHTSSHSSSSHHHSHHHHHHHHHTSHSSSSTSSSHSSSSSSSHSSSSSSSHSHSSSHHHHHHHSHSHSSTHTSSSSSSSSSSTSHHHHHHHSSSHHTSSTSSS
THTHHHSSSHSSTSSSSSSSHHHHHHHSTHTSSHHHHHHSSSSHTSSTSSSSSSSHHHHSSSHHTSHHHSSSHSSHSSSSSTSSSSTTSSSSHSTSSHHSSSHSTSSSSSSSHTSSHTSSHH
TSSSSSSSSSSSSSSHSSSHTTTHSSSSSSSHHSSSTSTSTTSSTSTSSSSSHSSSSHHSHSSSSTSHSSHSSSSHSTTSSSTSSSSSSSSSSSSSSHTHHHSHSSSSSSSTSTHHHHHHTSS
STHSHHTTHHTHHTHSSSSSHHTSSSTSSSSHSSSSSHSSHTTTSSSSSSSTDTSSSSTSSSSHTSSSSSTSSHTSSSSSHSSSSSSSSSSSSHSSSSSSSSHHHHHSTTSHHHHHHHHHH
TTHHHHHHHHHHHHHHHHHHHHHHHHTHSHSSSSSSHSTSTTTSSHHHHSSSHHSHSTSSHHHHHHHHHSSTDTHHHHHHHTSSSSSSHSSHHHHHHHHHHHHHHHHHHTHHHHHTSSSSS
SHHHHHHHHHHHHTSSHHHHHHTHHHHHHHHHHHHHHHHHHHHHSHHHTHHHHHHHHHHHHHHHHHHHHHHHSHTTSHSHSHHHHHHHSSHHHHHHHHHHHHHHHHHHHHHHHHHHHHHHHHH
HHHHHHHHHHHHHHHHHSSSHSSTHTTSTSSHSSSSSSSSTTSSSTSSSSSSSSTSSSSSSSHSSSHTTSSSSSHSTSSSSHTHSSSSSSHHSSTSSSSSHHHSSSSTSHHHSSTSSSSSSS
SHHHHHHHHHHTD

7xns_A

TQSYTNSFTRGVYYPDKVFRSSVLHSTQDLFLPFFSNVTWFHAIHVSGTNGTKRFDNPVLPFNDGVYFASTEKSNIIRGWIFGTTLDSKTQSLLIVNNATNVVIKVCEFQF
CNDPFLDVYYHKNNKSWMESEFRVYSSANNCTFEYVSQPFLMDLEGKQGNFKNLREFVFKNIDGYFKIYSKHTPINLGRDLPQGFSALEPLVDLPIGINITRFQTLLALHRSYLTPGDSSS
GWTAGAAAYYVGYLQPRTFLLKYNENGTITDAVDCALDPLSETKCTLKSFTVEKGIYQTSNFRVQPTESIVRFPNITNLCPFDEVFNATRFASVYAWNRKRISNCVADYSVLYNFAPFFAF
KCYGVSPTKLNDLCFTNVYADSFVIRGNEVSQIAPGQTGNIADYNYKLPDDFTGCVIAWNSNKLDSKVGGNYNYQYRLFRKSNLKPFERDISTEIYQAGNKPCNGVAGFNCYFPLRSYGFR
PTYGVGHQPYRVVVLSFELLHAPATVCGPKKSTNLVKNKCVNFNFNGLTGTGVLTESNKKFLPFQQFGRDIADTTDAVRDPQTLEILDITPCSFGGVSVITPGTNTSNQVAVLYQGVNCTE
VPVAIHADQLTPTWRVYSTGSNVFQTRAGCLIGAEYVNNSYECDIPIGAGICASYQTQXSQSIIAYTMSLGAENLVAYSNNSIAIPTNFTISVTTEILPVSMTKTSVDCTMYICGDSTECS
NLLLQYGSFCTQLKRALTGIAVEQDKNTQEVFAQVKQIYKTPPIKYFGGFNFSQILPDPSKPSKRSPIEDLLFNKVTLXLICAQKFNGLTVLPPLLTDEMIAQYTSALLAGTITSGWTFGA
GPALQIPFPMQMAYRFNGIGVTQNVLYENQKLIANQFNSAIGKIQDSLSSTPSALGKLQDVVNHNAQALNTLVKQLSSKFGAISSVLNDILSRLDPPEAEVQIDRLITGRLQSLQTYVTQQ
LIRAAEIRASANLAATKMSECVLGQSKRVDFCGKGYHLMSFPQSAPHGVVFLHVTYVPAQEKNFTTAPAICHDGKAHFPREGVFVSNGTHWFVTQRNFYEPQIITTDNTFVSGNCDVVIGI
VNNTVYDPLQPELDSFKEX

TTSSSSSHTSTSSSHSHSSSSTSSSSSSSSSSSSSTSSSSSSSSSSSTHHSTSSHTHTSSSHTSTTSSSSSTSHSTHSSTSSSTHHTSHHSSSSSTSSSHTSSSSSSSSSHS
SHSSSSSHHTSHHHSHSSSTTSSHSHSSSSSSTTSSSSSHSSSSSSSTTTSSSSSSSSSSSSTTSSSSSSSSSHSHTHSHTSSTSSSSSSHSSSSHTSSSHSSSSSSTHHSSSTSSSSHSS
TTSSTSHTSSSSSSSSTSSSSSSSSHHTSSHSSSSHHHSHHHHHHHHHTSHSSSSTSSSHSSSSSSSHSSSSSSSSSSTSSSHHHHHSSHSSSSHHSSSSSSSSTSSTSHHHHHHHSSSHHT
TTHHSSHHHHHHSSSHSSSSSSSSSSHHTHHHTSSTSSTHHHHHHSSSSHTSSSTSSSSSTHHHTSSHHTSHHSSSSHSSHSSSSSTSSSHSSSSSSTSHSSTHSHSTSTSSSHSSSSSSS
HHSSSTHSSSSSSSSSSSSSTSSSHSSSTSSSSSSSSTSSSSSSSTTSSSSTSSSSSTSTSSHHSHSSSSSTTSSHSSSSHHSTSSSSSSSSSSSSSSSSSSSSHHHSHSSSSSSSTSSHHH
HHHHTSTTHSHSHSHHHTTTTTSSSSHHTSSSTSSSSTSSSSSHSSHTTTSSSSSSSTDTSSSSSSSSSHTSSSSSTSSHTSSSSSHSSSSSSSSSSSSHSSSSSSSSHHHHHSTTSHHHH
HHHHHHTTHHHHHHHHHHHHHHHHHHHHHHHHHTHSHSSSSSSHSSSTTTSSHHHHSSSHHSHSTSSHHHHHHHHHSSTDTHHHHTHHTSSSSSSHSSHHHHHHHHHHHHHHHHHTHHHHH
SSSSSSSHHHHHHHHHHHHTSSHHHHHHTHHHHHHHHHHHHHHHHHHHHHSHHHTHHHHHHHHHHHHHHHHHHHHHHHHSHTTSHSHSHHHHHHHSSHHHHHHHHHHHHHHHHHHHHHHHHHHH
HHHHHHHHHHHHHHHHHHHHHHHHTSSHSSTHTTSTSSHSSSSSSSSTSSSSSSSSSSSHSSSSSSSSHSSSHTTSSSSSHSTSSSSHTHSSSSSSHHSSTSSSSSHHHSSSSTSTHHSSTS
SSSSSSSHHHHHHHHHHTD

7tnw_A

QCVNLTTRTQLPPAYTNSFTRGVYYPDKVFRSSVLHSTQDLFLPFFSNVTWFHVIXKRFDNPVLPFNDGVYFASIEKSNIIRGWIFGTTLDSKTQSLLIVNNATNVVIKVC
EFQFCNDPFFDHKNNKSWMESEFRVYSSANNCTFEYVSQPFLMDLEGKQGNFKNLREFVFKNIDGYFKIYSKHTPIIVREPEDLPQGFSALEPLVDLPIGINITRFQTLLALXAGAAAYYV
GYLQPRTFLLKYNENGTITDAVDCALDPLSETKCTLKSFTVEKGIYQTSNFRVQPTESIVRFPNITNLCPFDEVFNATRFASVYAWNRKRISNCVADYSVLYNLAPFFTFKCYGVSPTKLN
DLCFTNVYADSFVIRGDEVRQIAPGQTGNIADYNYKLPDDFTGCVIAWNSNKLDSKVSGNYNYLYRLFRKSNLKPFERDISTEIYQAGNKPCNGVAGFNCYFPLRSYSFRPTYGVGHQPYR
VVVLSFELLHAPATVCGPKKSTNLVKNKCVNFNFNGLKGTGVLTESNKKFLPFQQFGRDIADTTDAVRDPQTLEILDITPCSFGGVSVITPGTNTSNQVAVLYQGVNCTEVPVAIHADQLT
PTWRVYSTGSNVFQTRAGCLIGAEYVNNSYECDIPIGAGICASYQTQTKSXVASQSIIAYTMSLGAENSVAYSNNSIAIPTNFTISVTTEILPVSMTKTSVDCTMYICGDSTECSNLLLQY
GSFCTQLKRALTGIAVEQDKNTQEVFAQVKQIYKTPPIKYFGGFNFSQILPDPSKPSKRSFIEDLLFNKVTLADAGFIKQYGDCLGDIAARDLICAQKFKGLTVLPPLLTDEMIAQYTSAL
LAGTITSGWTFGAGAALQIPFAMQMAYRFNGIGVTQNVLYENQKLIANQFNSAIGKIQDSLSSTASALGKLQDVVNHNAQALNTLVKQLSSKFGAISSVLNDIFSRLDKVEAEVQIDRLIT
GRLQSLQTYVTQQLIRAAEIRASANLAATKMSECVLGQSKRVDFCGKGYHLMSFPQSAPHGVVFLHVTYVPAQEKNFTTAPAICHDGKAHFPREGVFVSNGTHWFVTQRNFYEPQIITTDN
TFVSGNCDVVIGIVNNTVYDPLQPELDSFKEELDKYFKNHTSPX

TTSSSHHSSSSSSSSSSSSHHSTSSSHSTSSSSTSSSSSSSSSSSSSTSSSSSSSSTDTSSSTSSSSTSTTSSSSSSSSSTTHSHTSSSSHHTTTHSSSSSSSSSHHSSSSSSS
TSSSSHTSSSSHTTTHHSSSHSTTHSHSTHSSHSSSSSSSSTSSSSTTSSTTSSSSSSSSSSSSTTSSSSSSSSSSSSSSHHSSTSSTSSSSSSHSSSTHTSSSHSSSSSSSTDTSTSSSSS
SSSSSSSSSSSSSHHTSSHSSSSHHHSHHHHHHHHHTSHSSSSTSSSSSSSSSSSSHSSSSSSSTSHSSSTHHHHHTSHSSSSHHTSSSSSSSSTSSSSHHHHHHHSHTTSSSSHHSSHHHHH
HSSSHSSSSSSSSSSHHHHHHSSTSSTHHHHHHSSSSHHSSTSSSSSSSHHHHHSSHHTTHHSSSSHSSHSSSSSSTSSSHSHSSSSHSHSTTSTHSSSTSSSHSSSSTTSHHSSSTHSSSS
SSSSTSSSTHSSSHSSSHSSSSSSSSTSSSSSSSTTTSSSSSSSSSHSTSSHHSHSSSSHHTSSHSSSSHHSTSSSSSSSSSSSTSSSSSSSSSHHHSHSSSSSSSSTSSHHHHHHSTHSSSH
SHTHHHHHTTHSSSSHHTSSSTSSSSHSSSSSHSSHTTTSSSSSSSSSTHTDTSSSSSSSSSSSSHTSSSSSTSSHHSSSSSHSSSSSSSSSSSSSHSSSSSSSSHHHHHTSTSHHHHHHHHHH
THHHHHHHHHHHHHHHHHHHHHHHHHTHSHSSSSSSHSTSTTTSTHHHHSSSHHSHSTSSHHHHHHHHHSSSSTHTSSSHTHHHSTHHHHHSHHHHHHHHTSSSSSSHSSHHHHHHHHHHH
HHHHHHHTHHHHHSSSSSSSHHHHHHHHHHHHTSSHHHHHHHHHHHHHHHHHHHHHHHHHHHHHHHSHTHHHHHHHHHHHHHHHHHHHHHHHHSHTTSHSHSHHHHHHHSSHHHHHHHHHHHHH
HHHHHHHHHHHHHHHHHHHHHHHHHHHHHHHHHHHHSSSHSSTHTTSTSSHSSSSSSSSTSSSSSSSSSSSHSSSSSSSSHSSSHTTSSSSSHSTSSSSHTHSSSSSSHHSSSSSSSSSHHH
SSSSTSHHHSSTSSSSSSSSHHHHHHHHHHHHHHHHHHHHHHTD

7to4_A

QCVNLTTRTQLPPAYTNSFTRGVYYPDKVFRSSVLHSTQDLFLPFFSNVTWFHVIXKRFDNPVLPFNDGVYFASIEKSNIIRGWIFGTTLDSKTQSLLIVNNATNVVIKVC
EFQFCNDPFFDHKNNKSWMESEFRVYSSANNCTFEYVSQPFLMDLEGKQGNFKNLREFVFKNIDGYFKIYSKHTPIIVREPEDLPQGFSALEPLVDLPIGINITRFQTLLALXAGAAAYYV
GYLQPRTFLLKYNENGTITDAVDCALDPLSETKCTLKSFTVEKGIYQTSNFRVQPTESIVRFPNITNLCPFDEVFNATRFASVYAWNRKRISNCVADYSVLYNLAPFFTFKCYGVSPTKLN
DLCFTNVYADSFVIRGDEVRQIAPGQTGNIADYNYKLPDDFTGCVIAWNSNKLDSKVSGNYNYLYRLFRKSNLKPFERDISTEIYQAGNKPCNGVAGFNCYFPLRSYSFRPTYGVGHQPYR
VVVLSFELLHAPATVCGPKKSTNLVKNKCVNFNFNGLKGTGVLTESNKKFLPFQQFGRDIADTTDAVRDPQTLEILDITPCSFGGVSVITPGTNTSNQVAVLYQGVNCTEVPVAIHAXWRV
YSTGSNVFQTRAGCLIGAEYVNNSYECDIPIGAGICASYQTQTKXVASQSIIAYTMSLGAENSVAYSNNSIAIPTNFTISVTTEILPVSMTKTSVDCTMYICGDSTECSNLLLQYGSFCTQ
LKRALTGIAVEQDKNTQEVFAQVKQIYKTPPIKYFGGFNFSQILPDPSKPSKRSFIEDLLFNKVTLADAGFIKQYGDCLGDIAARDLICAQKFKGLTVLPPLLTDEMIAQYTSALLAGTIT
SGWTFGAGAALQIPFAMQMAYRFNGIGVTQNVLYENQKLIANQFNSAIGKIQDSLSSTASALGKLQDVVNHNAQALNTLVKQLSSKFGAISSVLNDIFSRLDKVEAEVQIDRLITGRLQSL
QTYVTQQLIRAAEIRASANLAATKMSECVLGQSKRVDFCGKGYHLMSFPQSAPHGVVFLHVTYVPAQEKNFTTAPAICHDGKAHFPREGVFVSNGTHWFVTQRNFYEPQIITTDNTFVSGN
CDVVIGIVNNTVYDPLQPELDSFKEELDKYFKNHTSPX

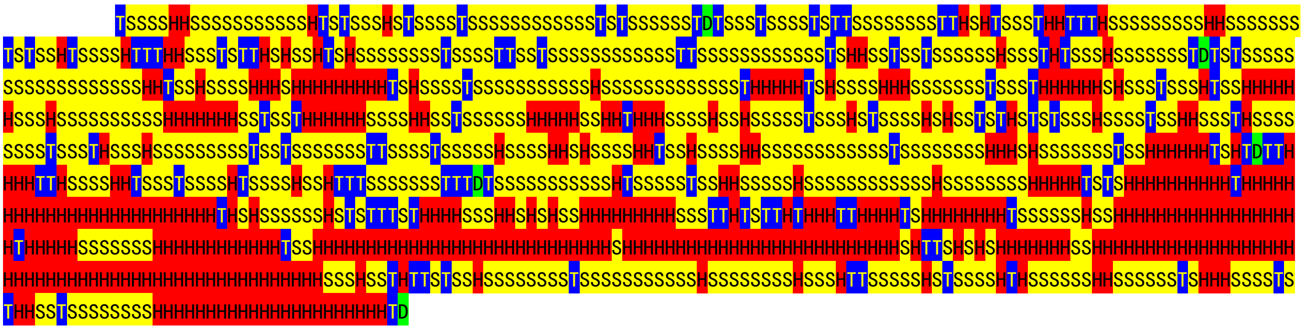

7to4_B

QCVNLTTRTQLPPAYTNSFTRGVYYPDKVFRSSVLHSTQDLFLPFFSNVTWFHVIXKRFDNPVLPFNDGVYFASIEKSNIIRGWIFGTTLDSKTQSLLIVNNATNVVIKVC
EFQFCNDPFFDHKNNKSWMESEFRVYSSANNCTFEYVSQPFLMDLEGKQGNFKNLREFVFKNIDGYFKIYSKHTPIIVREPEDLPQGFSALEPLVDLPIGINITRFQTLLALXAGAAAYYV
GYLQPRTFLLKYNENGTITDAVDCALDPLSETKCTLKSFTVEKGIYQTSNFRVQPTESIVRFPNITNLCPFDEVFNATRFASVYAWNRKRISNCVADYSVLYNLAPFFTFKCYGVSPTKLN
DLCFTNVYADSFVIRGDEVRQIAPGQTGNIADYNYKLPDDFTGCVIAWNSNKLDSKVSGNYNYLYRLFRKSNLKPFERDISTEIYQAGNKPCNGVAGFNCYFPLRSYSFRPTYGVGHQPYR
VVVLSFELLHAPATVCGPKKSTNLVKNKCVNFNFNGLKGTGVLTESNKKFLPFQQFGRDIADTTDAVRDPQTLEILDITPCSFGGVSVITPGTNTSNQVAVLYQGVNCTEVPVAIHADQLT
PTWRVYSTGSNVFQTRAGCLIGAEYVNNSYECDIPIGAGICASYQTQTKSXVASQSIIAYTMSLGAENSVAYSNNSIAIPTNFTISVTTEILPVSMTKTSVDCTMYICGDSTECSNLLLQY
GSFCTQLKRALTGIAVEQDKNTQEVFAQVKQIYKTPPIKYFGGFNFSQILPDPSKPSKRSFIEDLLFNKVTLADAGFIKQYGDCLGDIAARDLICAQKFKGLTVLPPLLTDEMIAQYTSAL
LAGTITSGWTFGAGAALQIPFAMQMAYRFNGIGVTQNVLYENQKLIANQFNSAIGKIQDSLSSTASALGKLQDVVNHNAQALNTLVKQLSSKFGAISSVLNDIFSRLDKVEAEVQIDRLIT
GRLQSLQTYVTQQLIRAAEIRASANLAATKMSECVLGQSKRVDFCGKGYHLMSFPQSAPHGVVFLHVTYVPAQEKNFTTAPAICHDGKAHFPREGVFVSNGTHWFVTQRNFYEPQIITTDN
TFVSGNCDVVIGIVNNTVYDPLQPELDSFKEELDKYFKNHTSPX

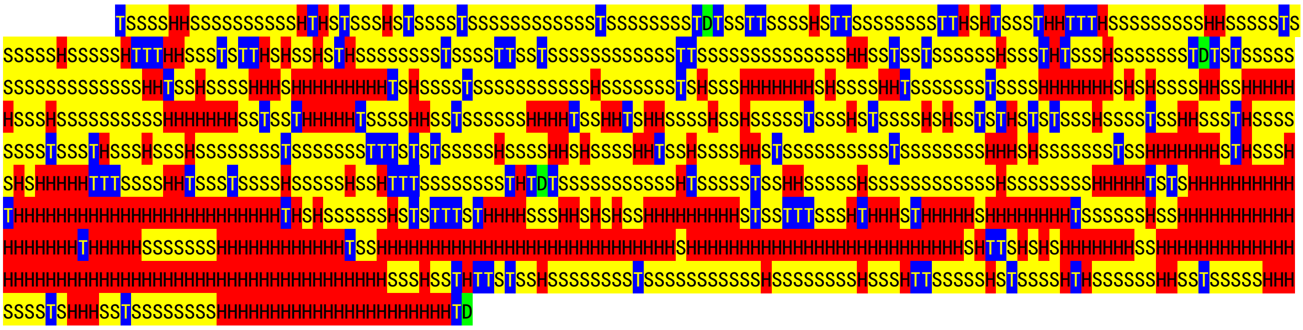

7krq_A

QCVNLTTRTQLPPAYTNSFTRGVYYPDKVFRSSVLHSTQDLFLPFFSNVTWFHAIHXKRFDNPVLPFNDGVYFASTEKSNIIRGWIFGTTLDSKTQSLLIVNNATNVVIKV
CEFQFCNDPFLGVYYHKNNKSWMESEFRVYSSANNCTFEYVSQPFLMDLEGKQGNFKNLREFVFKNIDGYFKIYSKHTPINLVRDLPQGFSALEPLVDLPIGINITRFQTLLALXSSSGWT
AGAAAYYVGYLQPRTFLLKYNENGTITDAVDCALDPLSETKCTLKSFTVEKGIYQTSNFRVQPTESIVRFPNITNLCPFGEVFNATRFASVYAWNRKRISNCVADYSVLYNSASFSTFKCY
GVSPTKLNDLCFTNVYADSFVIRGDEVRQIAPGQTGKIADYNYKLPDDFTGCVIAWNSNNLDSKVGGNYNYLYRLFRKSNLKPFERDISTEIYQAGSTPCNGVEGFNCYFPLQSYGFQPTN
GVGYQPYRVVVLSFELLHAPATVCGPKKSTNLVKNKCVNFNFNGLTGTGVLTESNKKFLPFQQFGRDIADTTDAVRDPQTLEILDITPCSFGGVSVITPGTNTSNQVAVLYQGVNCTEVPV
AIHADQLTPTWRVYSTGSNVFQTRAGCLIGAEHVNNSYECDIPIGAGICASYQTXSQSIIAYTMSLGAENSVAYSNNSIAIPTNFTISVTTEILPVSMTKTSVDCTMYICGDSTECSNLLL
QYGSFCTQLNRALTGIAVEQDKNTQEVFAQVKQIYKTPPIKDFGGFNFSQILPDPSKPSKRSFIEDLLFNKVTLADAGFIKQYGDCLGDIAARDLICAQKFNGLTVLPPLLTDEMIAQYTS
ALLAGTITSGWTFGAGAALQIPFAMQMAYRFNGIGVTQNVLYENQKLIANQFNSAIGKIQDSLSSTASALGKLQDVVNQNAQALNTLVKQLSSNFGAISSVLNDILSRLDKVEAEVQIDRL
ITGRLQSLQTYVTQQLIRAAEIRASANLAATKMSECVLGQSKRVDFCGKGYHLMSFPQSAPHGVVFLHVTYVPAQEKNFTTAPAICHDGKAHFPREGVFVSNGTHWFVTQRNFYEPQIITT
DNTFVSGNCDVVIGIVNNTVYDPLQPELDSFKEELDKYFKNHTSPX
TSSSSHTSSSSSSSSSSSTHHSTSSSHSTSSSSTSSSSSSSSSSSSSTSSSSSSSSSTDTSTSTSSSSTSTTSSSSSSSSTTHSHTSSSTHHTHTHSSSSSSSTSHHSSSSSS
STSSSSHSSSSSSSSTSTHTSSSSHSTSHSHSTHSSHSSSSSSSSSSSSHSTSSHSSHSSSSSSSSSSSTTSSSSSSSSSSSSTSHTSSSSTSSSSSSHSSSTHTSSSHSSSSSSSTDTHSTSS
SSSSSSSSSSSSSSSSSSSSSSHHTSSHSSSSHHHSHHHHHHHHHTSHSSSSTSSSSSSSSSSSSHSSSSSSSTSHSSSTHHHHHHSHSSSSHHTSSSSSSSSTSSSTHHHHHHSHHSHSTSSH
HSSHHHHHHSSSHSSSSSSSSSSHHHHHHHSSTSSTHHHHHHSSSSHTSSTSSSSSSHHHHHSSHHTTHHSSSSHSSHSSSSSTSSSHSHSSSSHSHSSSSTHSSSTSSSHSSSSTSSHHS
SSTHSSSSSSSSTSSSTHSSSHSSSTSSSSSSSSTSSSSSSSTTTSSSSSSSSSHSTSSHHSHSSSSHHTSSHSSSSHHSTSSSSSSSSSSSSSSSSSSSSHHHSHSSSSSSSTSSHHHHHH
HTHHSSSHSHTHHHHHTTTSSSSHHTSSSTSSSSHSSSSSHSSHTTTSSSSSSTDTSSSSSSSSSSHTSSSSSTSSHHSSSSSHSSSSSSSSSSSSHSSSSSSSSHHHHHTSTSHHHHHHHH
HHTHHHHHHHHHHHHHHHHHHHHHHHHHHTHSHSSSSSSHSTTTTTSTHHHHSSSHHSHSTSSHHHHHHHHHSSSSTHTTTSTTHHHSTHHHHTSHHHHHHHHTSSSSSSHSSHHHHHHHHH
HHHHHHHHHTHHHHHSSSSSSSHHHHHHHHHHHHTSSHHHHHHHHHHHHHHHHHHHHHHHHHHHHHSHHHHHHHHHHHHHHHHHHHHHHHHHHHHSHTTSHSHSHHHHHHHSSHHHHHHHHHHH
HHHHHHHHHHHHHHHHHHHHHHHHHHHHHHHHHHHHHHSSSHSSTHTTSTSSHSSSSSSSSTSSSSSSSSSSSSHTSSSSSSSHSSSHTTSSSSSHSTSSSSHTHSSSSSSTTSSSSSSSSSH
HHSSSSTSHHHSSTSSSSSSSSHHHHHHHHHHHHHHHHHHHHHHTD

7krq_B

QCVNLTTRTQLPPAYTNSFTRGVYYPDKVFRSSVLHSTQDLFLPFFSNVTWFHAIHXKRFDNPVLPFNDGVYFASTEKSNIIRGWIFGTTLDSKTQSLLIVNNATNVVIKV
CEFQFCNDPFLGVYYHKNNKSWMESEFRVYSSANNCTFEYVSQPFLMDLEGKQGNFKNLREFVFKNIDGYFKIYSKHTPINLVRDLPQGFSALEPLVDLPIGINITRFQTLLALXSSSGWT
AGAAAYYVGYLQPRTFLLKYNENGTITDAVDCALDPLSETKCTLKSFTVEKGIYQTSNFRVQPTESIVRFPNITNLCPFGEVFNATRFASVYAWNRKRISNCVADYSVLYNSASFSTFKCY
GVSPTKLNDLCFTNVYADSFVIRGDEVRQIAPGQTGKIADYNYKLPDDFTGCVIAWNSNNLDSKVGGNYNYLYRLFRKSNLKPFERDISTEIYQAGSTPCNGVEGFNCYFPLQSYGFQPTN
GVGYQPYRVVVLSFELLHAPATVCGPKKSTNLVKNKCVNFNFNGLTGTGVLTESNKKFLPFQQFGRDIADTTDAVRDPQTLEILDITPCSFGGVSVITPGTNTSNQVAVLYQGVNCTEVPV
AIHADQLTPTWRVYSTGSNVFQTRAGCLIGAEHVNNSYECDIPIGAGICASYQTXSQSIIAYTMSLGAENSVAYSNNSIAIPTNFTISVTTEILPVSMTKTSVDCTMYICGDSTECSNLLL
QYGSFCTQLNRALTGIAVEQDKNTQEVFAQVKQIYKTPPIKDFGGFNFSQILPDPSKPSKRSFIEDLLFNKVTLADAGFIKQYGDCLGDIAARDLICAQKFNGLTVLPPLLTDEMIAQYTS
ALLAGTITSGWTFGAGAALQIPFAMQMAYRFNGIGVTQNVLYENQKLIANQFNSAIGKIQDSLSSTASALGKLQDVVNQNAQALNTLVKQLSSNFGAISSVLNDILSRLDKVEAEVQIDRL
ITGRLQSLQTYVTQQLIRAAEIRASANLAATKMSECVLGQSKRVDFCGKGYHLMSFPQSAPHGVVFLHVTYVPAQEKNFTTAPAICHDGKAHFPREGVFVSNGTHWFVTQRNFYEPQIITT
DNTFVSGNCDVVIGIVNNTVYDPLQPELDSFKEELDKYFKNHTSPX
TSSSSHTSSSSSSSSSSSTHHSTSSSHSTSSSSTSSSSSSSSSSSSSTSSSSSSSSSTDTSTSTSSSSTSTTSSSSSSSSTTHSHTSSSTHHTHTHSSSSSSSSSHHSSSSSS
STSSSSHSSSSSSSSTTTHTSSSSHSTSHSHSTHSSHSSSSSSSSSSSSHSTSSHSSHSSSSSSSSSSSTTSSSSSSSSSSSSTSHTSSSSTSSSSSSHSSSTHTSSSHSSSSSSSTDTHSTSS
SSSSSSSSSSSSSSSSSSSSSSHHTSSHSSSSHHHSHHHHHHHHHTSHSSSSTSSSSSSSSSSSSHSSSSSSSTSHSSSTHHHHHHSHSSSSHHTSSSSSSSSTSSSTHHHHHHSHHSHSTSSH
HSSHHHHHHSSSHSSSSSSSSSSHHHHHHHSSTSSTHHHHHHSSSSHTSSTSSSSSSHHHHHSSHHTTHHSSSSHSSHSSSSSTSSSHSHSSSSHSHSSSSTHSSSTSSSHSSSSTSSHHS
SSTHSSSSSSSSTSSSTHSSSHSSSTSSSSSSSSTSSSSSSSTTTSSSSSSSSSHSTSSHHSHSSSSHHTSSHSSSSHHSTSSSSSSSSSSSSSSSSSSSSHHHSHSSSSSSSTSSHHHHHH
HSHHSSSHSHTHHHHHTTTSSSSHHTSSSTSSSSHSSSSSHSSHTTTSSSSSSTDTSSSSSSSSSSHTSSSSSTSSHHSSSSSHSSSSSSSSSSSSHSSSSSSSSHHHHHTSTSHHHHHHHH
HHTHHHHHHHHHHHHHHHHHHHHHHHHHHTHSHSSSSSSHSTTTTTSTHHHHSSSHHSHSTSSHHHHHHHHHSSSSTHTTTSTTHHHSTHHHHTSHHHHHHHHTSSSSSSHSSHHHHHHHHH
HHHHHHHHHTHHHHHSSSSSSSHHHHHHHHHHHHTSSHHHHHHHHHHHHHHHHHHHHHHHHHHHHHSHHHHHHHHHHHHHHHHHHHHHHHHHHHHSHTTSHSHSHHHHHHHSSHHHHHHHHHHH
HHHHHHHHHHHHHHHHHHHHHHHHHHHHHHHHHHHHHHSSSHSSTHTTSTSSHSSSSSSSSTSSSSSSSSSSSSHTSSSSSSSHSSSHTTSSSSSHSTSSSSHTHSSSSSSTTSSSSSSSSSH
HHSSSSTSHHHSSTSSSSSSSSHHHHHHHHHHHHHHHHHHHHHHTD

6x6p_A

AYTNSFTRGVYYPDKVFRSSVLHSTQDLFLPFFSNVTWFHAIXDNPVLPFNDGVYFASTEKSNIIRGWIFGTTLDSKTQSLLIVNNATNVVIKVCEFQFCNDPFLGVXMES
EFRVYSSANNCTFEYVSXKNLREFVFKNIDGYFKIYSKHTPINXLPQGFSALEPLVDLPIGINITRFQTLLALHXAAYYVGYLQPRTFLLKYNENGTITDAVDCALDPLSETKCTLKSFTV
EKGIYQTSNFRVQPTESIVRFPNITNLCPFGEVFNATRFASVYAWNRKRISNCVADYSVLYNSASFSTFKCYGVSPTKLNDLCFTNVYADSFVIRGDEVRQIAPGQTGKIADYNYKLPDDF
TGCVIAWNSNNLDSKVGGNYNYLYRLFRKSNLKPFERDISTEIYQAGXNCYFPLQSYGFQPTNGVGYQPYRVVVLSFELLHAPATVCGPKKSTNLVKNKCVNFNFNGLTGTGVLTESNKKF
LPFQQFGRDIADTTDAVRDPQTLEILDITPCSFGGVSVITPGTNTSNQVAVLYQDVNCTEVPVAIHADQLTPTWRVYSTGSNVFQTRAGCLIGAEHVNNSYECDIPIGAGICASYQTXSQS
IIAYTMSLGAENSVAYSNNSIAIPTNFTISVTTEILPVSMTKTSVDCTMYICGDSTECSNLLLQYGSFCTQLNRALTGIAVEQDKNTQEVFAQVKQIYKTPPIKDFGGFNFSQILPDPSKP
SKRSFIEDLLFNKVTXFNGLTVLPPLLTDEMIAQYTSALLAGTITSGWTFGAGAALQIPFAMQMAYRFNGIGVTQNVLYENQKLIANQFNSAIGKIQDSLSSTASALGKLQDVVNQNAQAL
NTLVKQLSSNFGAISSVLNDILSRLDPPEAEVQIDRLITGRLQSLQTYVTQQLIRAAEIRASANLAATKMSECVLGQSKRVDFCGKGYHLMSFPQSAPHGVVFLHVTYVPAQEKNFTTAPA
ICHDGKAHFPREGVFVSNGTHWFVTQRNFYEPQIITTDNTFVSGNCDVVIGIVNNTVYDPLQPELDSX
TSSSSHTSTSSSHSHSSSSTSTSSSSSSSSSSTSTSSSSSTTDTTSSSSTHTTSSTSSSSHHTHSHSSSSSHHTSHHSSSSSSSSHTHSSSSSHSSSSSSHTSSSSTDTSH
SSHHSHSSHSTHSSSSTDTSSSSSSSSSSTTSSSSSSSSSSSTDTSTSSSSSSSHSSSTHTSTSHSSSSSSSSTDTSSSSSSSSSSSSSSSSSHHTSSHSSSSHHHSHHHHHHHHHTSHSS
SSTSSSHSSSSSSSHSSSSSSSSSHSSSTHHHHHTSHSSSSHHTSSSSSSSTSSSSHHHHHHHTHSHSSSSSTSSHHHHHTSSSHSSSSSSSSSSHHHHHHSSSTSSTHHHHHHSSSSHTS
TSSSSSSSTHHHHSSSTTSHHSSSSHSSHSSSSSTSSSHSHSSSSHTDTSSSHSSSSSSSHHSSHHTSSSSSSSSSSTSSHSSSHSSSSSSSSSSSSTSSSSSSSTTSSSSTSSSSSHSTS
SHHSHSSSSHHTSSHSSSSHHSTSSSSSSSSSSSSSSSSSSSHHHSHSSSSSSSTSSTHHHHHHHHHTHSHHTSHSSSSSHHSSSSHHTSSSTSSSSHSSSSSHSSHTTTSSSSSSTDTSS
SSSSSSSHTSSSSSTSSHHSSSSSHSSSSSSSSSSSSHSSSTSSSTHHHHHTSTSHHHHHHHHHHTHHHHHHHHHHHHHHHHHHHHHHHHHTHSHSSSSSSHSTTTTTSSHHHHSSSHHSH
STSSHHHHHHHHHHTDTTTTSSSSSHSSHHHHHHHHHHHHHHHHHHTHHHHHSSSSSSSHHHHHHHHHHHHTSSHHHHHHHHHHHHHHHHHHHHHHHHHHHHSHHHHHHHHHHHHHHHHHH
HHHHHHHHSHTTSHSHSHHHHHHHSSHHHHHHHHHHHHHHHHHHHHHHHHHHHHHHHHHHHHHHHHHHHHHHHHHHHHHHHSSSHSSTHHSSTSSHSSSSSSSSTTSSSSSSSSSSHSSSSSSSSHS
SSHTTSSSSSHSTSSSSHTHSSSSSSHHSSSSSSSSHHHSSSSTSHHHSSTSSSSSSSSHHHHHHHTD

6x6p_B

AYTNSFTRGVYYPDKVFRSSVLHSTQDLFLPFFSNVTWFHAIXDNPVLPFNDGVYFASTEKSNIIRGWIFGTTLDSKTQSLLIVNNATNVVIKVCEFQFCNDPFLGVXMES
EFRVYSSANNCTFEYVSXKNLREFVFKNIDGYFKIYSKHTPINXLPQGFSALEPLVDLPIGINITRFQTLLALHXAAYYVGYLQPRTFLLKYNENGTITDAVDCALDPLSETKCTLKSFTV
EKGIYQTSNFRVQPTESIVRFPNITNLCPFGEVFNATRFASVYAWNRKRISNCVADYSVLYNSASFSTFKCYGVSPTKLNDLCFTNVYADSFVIRGDEVRQIAPGQTGKIADYNYKLPDDF
TGCVIAWNSNNLDSKVGGNYNYLYRLFRKSNLKPFERDISTEIYQAGXNCYFPLQSYGFQPTNGVGYQPYRVVVLSFELLHAPATVCGPKKSTNLVKNKCVNFNFNGLTGTGVLTESNKKF
LPFQQFGRDIADTTDAVRDPQTLEILDITPCSFGGVSVITPGTNTSNQVAVLYQDVNCTEVPVAIHADQLTPTWRVYSTGSNVFQTRAGCLIGAEHVNNSYECDIPIGAGICASYQTXSQS
IIAYTMSLGAENSVAYSNNSIAIPTNFTISVTTEILPVSMTKTSVDCTMYICGDSTECSNLLLQYGSFCTQLNRALTGIAVEQDKNTQEVFAQVKQIYKTPPIKDFGGFNFSQILPDPSKP
SKRSFIEDLLFNKVTXFNGLTVLPPLLTDEMIAQYTSALLAGTITSGWTFGAGAALQIPFAMQMAYRFNGIGVTQNVLYENQKLIANQFNSAIGKIQDSLSSTASALGKLQDVVNQNAQAL
NTLVKQLSSNFGAISSVLNDILSRLDPPEAEVQIDRLITGRLQSLQTYVTQQLIRAAEIRASANLAATKMSECVLGQSKRVDFCGKGYHLMSFPQSAPHGVVFLHVTYVPAQEKNFTTAPA
ICHDGKAHFPREGVFVSNGTHWFVTQRNFYEPQIITTDNTFVSGNCDVVIGIVNNTVYDPLQPELDSX
TSSSSHTSTSSSHSHSSSSTSTSSSSSSSSSSTSTSSSSSTTDTTSSSSTHTTSSTSSSSHHTHSHSSSSSHHTSHHSSSSSSSSHTHSSSSSHSSSSSSHTSSSSTDTSH
SSHHSHSSHSTHSSSSTDTSSSSSSSSSSTTSSSSSSSSSSSTDTSTSSSSSSSHSSSTHTSTSHSSSSSSSSTDTSSSSSSSSSSSSSSSSSHHTSSHSSSSHHHSHHHHHHHHHTSHSS
SSTSSSHSSSSSSSHSSSSSSSSSHSSSTHHHHHTSHSSSSHHTSSSSSSSTSSSSHHHHHHHTHSHSSSSSTSSHHHHHTSSSHSSSSSSSSSSHHHHHHSSSTSSTHHHHHHSSSSHTS
TSSSSSSSTHHHHSSSTTSHHSSSSHSSHSSSSSTSSSHSHSSSSHTDTSSSHSSSSSSSHHSSHHTSSSSSSSSSSTSSHSSSHSSSSSSSSSSSSTSSSSSSSTTSSSSTSSSSSHSTS
SHHSHSSSSHHTSSHSSSSHHSTSSSSSSSSSSSSSSSSSSSHHHSHSSSSSSSTSSTHHHHHHHHHTHSHHTSHSSSSSHHSSSSHHTSSSTSSSSHSSSSSHSSHTTTSSSSSSTDTSS
SSSSSSSHTSSSSSTSSHHSSSSSHSSSSSSSSSSSSHSSSTSSSTHHHHHTSTSHHHHHHHHHHTHHHHHHHHHHHHHHHHHHHHHHHHHTHSHSSSSSSHSTTTTTSSHHHHSSSHHSH
STSSHHHHHHHHHHTDTTTTSSSSSHSSHHHHHHHHHHHHHHHHHHTHHHHHSSSSSSSHHHHHHHHHHHHTSSHHHHHHHHHHHHHHHHHHHHHHHHHHHHSHHHHHHHHHHHHHHHHHH
HHHHHHHHSHTTSHSHSHHHHHHHSSHHHHHHHHHHHHHHHHHHHHHHHHHHHHHHHHHHHHHHHHHHHHHHHHHHHHHHHSSSHSSTHHSSTSSHSSSSSSSSTTSSSSSSSSSSHSSSSSSSSHS
SSHTTSSSSSHSTSSSSHTHSSSSSSHHSSSSSSSSHHHSSSSTSHHHSSTSSSSSSSSHHHHHHHTD

7jjc_G NSPRRARX TSSHTSTD

7xix_A

TQSYTNSFTRGVYYPDKVFRSSVLHSTQDLFLPFFSNVTWFHAIHVSGTNGTKRFDNPVLPFNDGVYFASTEKSNIIRGWIFGTTLDSKTQSLLIVNNATNVVIKVCEFQF
CNDPFLDVYYHKNNKSWMESEFRVYSSANNCTFEYVSQPFLMDLEGKQGNFKNLREFVFKNIDGYFKIYSKHTPINLGRDLPQGFSALEPLVDLPIGINITRFQTLLALHRSYLTPGDSSS
GWTAGAAAYYVGYLQPRTFLLKYNENGTITDAVDCALDPLSETKCTLKSFTVEKGIYQTSNFRVQPTESIVRFPNITNLCPFDEVFNATRFASVYAWNRKRISNCVADYSVLYNFAPFFAF
KCYGVSPTKLNDLCFTNVYADSFVIRGNEVSQIAPGQTGNIADYNYKLPDDFTGCVIAWNSNKLDSKVGGNYNYLYRLFRKSNLKPFERDISTEIYQAGNKPCNGVAGFNCYFPLRSYGFR
PTYGVGHQPYRVVVLSFELLHAPATVCGPKKSTNLVKNKCVNFNFNGLTGTGVLTESNKKFLPFQQFGRDIADTTDAVRDPQTLEILDITPCSFGGVSVITPGTNTSNQVAVLYQGVNCTE
VPVAIHADQLTPTWRVYSTGSNVFQTRAGCLIGAEYVNNSYECDIPIGAGICASYQTQXSQSIIAYTMSLGAENSVAYSNNSIAIPTNFTISVTTEILPVSMTKTSVDCTMYICGDSTECS
NLLLQYGSFCTQLKRALTGIAVEQDKNTQEVFAQVKQIYKTPPIKYFGGFNFSQILPDPSKPSKRSPIEDLLFNKVTLXLICAQKFNGLTVLPPLLTDEMIAQYTSALLAGTITSGWTFGA
GPALQIPFPMQMAYRFNGIGVTQNVLYENQKLIANQFNSAIGKIQDSLSSTPSALGKLQDVVNHNAQALNTLVKQLSSKFGAISSVLNDILSRLDPPEAEVQIDRLITGRLQSLQTYVTQQ
LIRAAEIRASANLAATKMSECVLGQSKRVDFCGKGYHLMSFPQSAPHGVVFLHVTYVPAQEKNFTTAPAICHDGKAHFPREGVFVSNGTHWFVTQRNFYEPQIITTDNTFVSGNCDVVIGI
VNNTVYDPLQPELDSFKEX

TSSSSSSHTSTSSSHSTSSSSTSSSSSSSSSSSSTSSSSSSSSSSSTHHSTSSHTHTSSSHTSTTSSSSSTSHSTHSSTSSSTHHTSHHSSSSTSSSSHHSSSSSSSTSHS
THSSSSSHHTSHHHSHSSSTTSSHSHSSHSSSTSTSSSSHSSSSSSSTTTSSSSSSSSSSSSSTTSSSSSSSSTSHSHTHSHSSSTSSSSSSHSSSTHTSSSHSSSSTSSHHSSSTTSSSHSS
TTSSSSHTSSSSSSSTSSSSSSSSHHTSSHSSSSHHHSHHHHHHHHHTSHSSSSTSSSHSSSSSSSHSSSSSSSSSHSSSTHHHHHTSHSSSSHHTSSSSSSSTSSSSHHHHHHHSTSHHT
SSHHSSHHHHHHSSSHSSSSSSSSSSSHHHHHHTSSTSSTHHHHHHSSSSHTSSSSSSSSSTHHHHSSHHTSHHSSSSHSSHSSSSSTSSSHSTSSSSTTHSSTHSHSTSTSSSHSSSSTSS
HHSSSTHSSSSSSSSSSSTSSSSSHSSSTSSSSSSSSSTSSSSSSSTTSSSSTSSSSSSTSSSSHHSHSSSSSTTSSHSSSSHHSTSSSSSSSSSSSSSSSSSSSHHHSHSSSSSSSTSSHHH
HHHHSSSTHSHSHSHHHTTTTTSSSSHHTSSSTSSSSTSSSSSHSSHTTTSSSSSSSTDTSSSSSSSSSHTSSSSSSSSHTSSSSSHSSSSSSSSSSSSHSSSSSSSSHHHHHSTTSHHHH
HHHHHHTTHHHHHHHHHHHHHHHHHHHHHHHHTHSHSSSSSSHSSSTTTSSHHHHSSSHHSHSTSSHHHHHHHHHSSTDTHHHHTHHTSSSSSSHSSHHHHHHHHHHHHHHHHHHTHHHHH
SSSSSSSHHHHHHHHHHHHTSSHHHHHHTHHHHHHHHHHHHHHHHHHHHHSHTHTHHHHHHHHHHHHHHHHHHHHHHHSHTTSHSHSHHHHHHHSSHHHHHHHHHHHHHHHHHHHHHHHHHHH
HHHHHHHHHHHHHHHHHHHHHHHHSSSHSSTHTTSTSSHSSSSSSSSTSSSSSSSSSSSSSSSSSSSSSHSSSHTTSSSSSHSTSSSSHTHSSSSSSHHSSTSSSSSHHHSSSSTSHTHSSTS
SSSSSSSHHHHHHHHHHTD

7x6a_A

TQSYTNSFTRGVYYPDKVFRSSVLHSTQDLFLPFFSNVTWFHAIHVSGTNGTKRFDNPVLPFNDGVYFASTEKSNIIRGWIFGTTLDSKTQSLLIVNNATNVVIKVCEFQF
CNDPFLDVYYHKNNKSWMESEFRVYSSANNCTFEYVSQPFLMDLEGKQGNFKNLREFVFKNIDGYFKIYSKHTPINLGRDLPQGFSALEPLVDLPIGINITRFQTLLALHRSYLTPGDSSS
GWTAGAAAYYVGYLQPRTFLLKYNENGTITDAVDCALDPLSETKCTLKSFTVEKGIYQTSNFRVQPTESIVRFPNITNLCPFDEVFNATRFASVYAWNRKRISNCVADYSVLYNFAPFFAF
KCYGVSPTKLNDLCFTNVYADSFVIRGNEVSQIAPGQTGNIADYNYKLPDDFTGCVIAWNSNKLDSKVGGNYNYLYRLFRKSNLKPFERDISTEIYQAGNKPCNGVAGFNCYFPLRSYGFR
PTYGVGHQPYRVVVLSFELLHAPATVCGPKKSTNLVKNKCVNFNFNGLTGTGVLTESNKKFLPFQQFGRDIADTTDAVRDPQTLEILDITPCSFGGVSVITPGTNTSNQVAVLYQGVNCTE
VPVAIHADQLTPTWRVYSTGSNVFQTRAGCLIGAEYVNNSYECDIPIGAGICASYQTQXSQSIIAYTMSLGAENSVAYSNNSIAIPTNFTISVTTEILPVSMTKTSVDCTMYICGDSTECS
NLLLQYGSFCTQLKRALTGIAVEQDKNTQEVFAQVKQIYKTPPIKYFGGFNFSQILPDPSKPSKRSPIEDLLFNKVTLXLICAQKFNGLTVLPPLLTDEMIAQYTSALLAGTITSGWTFGA
GPALQIPFPMQMAYRFNGIGVTQNVLYENQKLIANQFNSAIGKIQDSLSSTPSALGKLQDVVNHNAQALNTLVKQLSSKFGAISSVLNDILSRLDPPEAEVQIDRLITGRLQSLQTYVTQQ
LIRAAEIRASANLAATKMSECVLGQSKRVDFCGKGYHLMSFPQSAPHGVVFLHVTYVPAQEKNFTTAPAICHDGKAHFPREGVFVSNGTHWFVTQRNFYEPQIITTDNTFVSGNCDVVIGI
VNNTVYDPLQPELDSFKEX

TSSSSSTHTSTSSSHSHSSSSTSSSSSSSSSSSSTSSSSSSSSSSSTHHSSSSHTHTSSSHTSTTSSSSSSSHHHHSSTSSSTHHTSHHSSSSTSSSSHTSSSSSSSTSHS
SHSSSSHTHTSHHHSTSSSTHTTHSHSSSSSTSSTSSSSHSSSSSSSTTTTSSSSSSSSSSSSTTSSSSSSSSSSSTTHHSHSSSTSSSSSSHSSSTHTTTSTSSSSTSSHHSSSTTSSSTSS
TSSSSSHTSSSSSSSTSSSSSSSSHHTSSHSSSSHHHSHHHHHHHHHTSHSSSSTSSSHSSSSSSSHSSSSSSSSSHSSSTHHHHHTSHSSSSHHTSSSSSSSTSSSSHHHHHHHSTSHHT
SSHHSSHHTHHHSSSHSSSSSSSSSSSHHHHHHSSSTSSTHHHHHHSSSSHTSSTSSSSSSSTHHHTSSHHTSHHSSSSHSSHSSSSSTSSSHSSSSSSTSHSSSHTTSTSTSSSHSSSSTSS
HHSSSTHSSSSSSSSSSSTSTSSSHSSSTSSSSSSSSSTSSSTSSSTTTSTSTSSSSSHSTSSHHSHSSSSTHTSSHSSSSHHHTSSSSSSSSSSTSSSSSSSSSHHHSHSSSSSSSTSSTHS
HHHTSSSTHSHSHSHHHTHTTTSSSSHHTSSSTSSSSTSSSSSHSSHTTTSSSSSSSTDTSSSSSSSSSHTSSSSSSSSHTSSSSSHSSSSSSSSSSSSHSSSSSSSSHHHHHSTTSHHHH
HHHHHHTTHHHHHHHHHHHHHHHHHHHHHHHHTHSHSSSSSSHSTSTTTSSHHHHSSSHHSHSTSSHHHHHHHHHSSTDTHHHHTHHTSSSSSSHSSHHHHHHHHHHHHHHHHHHTHHHHH
SSSSSSSHHHHHHHHHHHHTSSHHHHHHTHHHHHHHHHHHHHHHHHHHHHSHHHTHHHHHHHHHHHHHHHHHHHHHHHTSHTTSHSHSHHHHHHHSSHHHHHHHHHHHHHHHHHHHHHHHHHHH
HHHHHHHHHHHHHHHHHHHHHHHHSSSHSSTHTTSTSSHSSSSSSSSTSSSSSSSSSSSSSSSSSSSSSHSSSHTTSSSSSHSTSSSSHTHSSSSSSHHSSTSSSSSHHHSSSSTSHTHSSTS
SSSSSSSHHHHHHHHHHTD

7e9t_A

NSVAYSNNSIAIPTNFTISVTTEILPVSMTKTSVDCTMYICGDSTECSNLLLQYGSFCTQLNRALTGIXTQNVLYENQKLIANQFNSAIGKIQDSLSSTASALGKLQDVVN
QNAQALNTLVKQLSSNFGAISSVLNDILSRLDKVEAEVQIDRLITGRLQSLQTYVTQQLIRAAEIRASANLAATKMSECVLGQSKRVDFCGKGYHLMSFPQSAPHGVVFLHVTYVPAQEKN
FTTAPAICHDGKAHFPREGVFVSNGTHWFVTQRNFYEPQIITTDNTFVSGNCDVVIGIVNNTVYDPLQPELDSFKEELDKYFKNHTSPDVDLGDISGINXQKEIDRLNEVAKNLNESLXWP
WYIWLGFIAGLIAIVMVTIMLX

TSSHHSSSSSSSSHSSSSSSSSSSSSSHSSSSTSSSHHHHHHTHSHHHHHHHHHHTHHHHHHHHHHHSTDTHHHHHHHHHHHHHHHHHHHHHHHHHHHHHHHHHHHHHHHHHH
HHHHHHHHHHHHHHHHHHHHHHHHHHHHHHHHHHHHHHHHHHHHHHHHHHHHHHHHHHHHHHHHHHHHHHHHHHHHHHHHSSSHSSTSTTSTSSHSSSSSSSHHSSSSSSSSSSSHSSSS
SSSSHSSSSTTSSSSSSTSSTSSSHHSSSTSSHHSSTSSSSSHHHSSSSSTTSSSSSSSSSTSSSSSSSSTSSSSHHHHHHHHSSTSSSSSSSSSSTHHHHTDTHHHHHHHHHHHHHHHHTTDTH
HHHHHHHHHHHHHHHHHHHHTD

7acs_A

GAMGLPNNTASWFTALTQHGKEDLKFPRGQGVPINTNSSPDDQIGYYRRATRRIRGGDGKMKDLSPRWYFYYLGTGPEAGLPYGANKDGIIWVATEGALNTPKDHIGTRNP
ANNAAIVLQLPQGTTLPKGFYAEGSRGGSX

TSSTSTSHTSSHSSSSSSTSHSHSSSSSTSTSSTTHHSSHHHTSSSSSSSSSSSSSSHHTSSHTSSSSSSSSSSSTSTHTHTSSSTSSSSTSSSSSSSSTSSSSSTHHHTSTSH
HHSSSSSSSSSSTSSSSSTSSSSTSSTTTD

7act_A

GLPNNTASWFTALTQHGKEDLKFPRGQGVPINTNSSPDDQIGYYRRATRRIRGGDGKMKDLSPRWYFYYLGTGPEAGLPYGANKDGIIWVATEGALNTPKDHIGTRNPANN
AAIVLQLPQGTTLPKGFYAEGSRGGSX

TSSSHTSSHSSSSSSHSHSSTSSSTTSTSSSSHTSSHHHTSTSSSSSHTHTHTTTTSSHTSSSSSSSSSSTSTHHHHSSSTSTSSTSSSSSSHHSSHSSTHHHHHSSHHHS
SSSSSSSSTTSSSSHHSSSHSTSTTTD

6wkp_A ASWFTALTQHGKEDLKFPRGQGVPINTNSSPDDQIGYYRRAXPRWYFYYLGTGPEAGLPYGANKDGIIWVATEGALNTPKDHIGTRXAIVLQLPQGTTLPKGFYAX

TSHSSSSSSSTHSSSSSSSTSTSSSSHTSSHHHTSTSSSSTDTSSSSSSSTSTHTHHSSSTSTSSTSSSSSSSTSSTSSTHHHTSTDTSSSSSSSTSSSSSTSSTD

6wkp_B

NTASWFTALTQHGKEDLKFPRGQGVPINTNSSPDDQIGYYRRATRXKDLSPRWYFYYLGTGPEAGLPYGANKDGIIWVATEGALNTPKDHIGTRNPANNAAIVLQLPQGTT
LPKGFYAX

TTHSHSSSSSSHSHSSSSSSSTSTSSSSHTSSHHHTSTSSSSSSTDTSSSSSSSSSSSTSTHTHHSSSTSSSSTSSSSSSSTSSTSSTHHHTSSSHHHSSSSSSSSSSTSS
SSSTSSTD

6vyo_A

ASWFTALTQHGKEDLKFPRGQGVPINTNSSPDDQIGYYRRATRRIRGGDGKMKDLSPRWYFYYLGTGPEAGLPYGANKDGIIWVATEGALNTPKDHIGTRNPANNAAIVLQ
LPQGTTLPKGFYAX

TSHSSSSSSHSHSSSSSSSTSTSSSSHTSSHHHTSTSSSSSSSSSSSHHTSSSSSSSSSSSSSSTSTHTHHSSSTSSSSTSSSSSSSTSSTSSTHHHTSSSHHHSSSSSSS
SSSTSSSSSTSSTD

6vyo_B

TASWFTALTQHGKEDLKFPRGQGVPINTNSSPDDQIGYYRRATRRIRGGDGKMKDLSPRWYFYYLGTGPEAGLPYGANKDGIIWVATEGALNTPKDHIGTRNPANNAAIVL
QLPQGTTLPKGFYAX

TSSHSSSSSSHSHSSSSSSSTSTSSSSHHSSHHHTSTSSSSSSSSSSSHHTSSSSSSSSSSSSSSTSTHTHHSSSTSSSSTSSSSSSSTSSTSSTHHHTSSSHHHSSSSSS
SSSHHSSSSSTSSTD

6vyo_C

TASWFTALTQHGKEDLKFPRGQGVPINTNSSPDDQIGYYRRATRRIRGGDGKMKDLSPRWYFYYLGTGPEAGLPYGANKDGIIWVATEGALNTPKDHIGTRNPANNAAIVL
QLPQGTTLPKGFYAX

TSSHSSSSSSHSHSSSSSSSTSTSSSSHTSSHHHTSTSSSSSSSSSSSHHTSSSSSSSSSSSSSSTSTHTHHSSSTSSSSTSSSSSSSTSSTSSTHHHTSSSHHHSSSSSS
SSSTHSSSSSTSSTD

6vyo_D

TASWFTALTQHGKEDLKFPRGQGVPINTNSSPDDQIGYYRRATRRIRGGDGKMKDLSPRWYFYYLGTGPEAGLPYGANKDGIIWVATEGALNTPKDHIGTRNPANNAAIVL
QLPQGTTLPKGFYAX

TSSHSSSSSSHSHSSSSSSSTSTSSSTHHSSHHHTSTSSSSSSSSSSSHHTSSSSSSSSSSSSSSTSTHTHHSSSTSSSSTSSSSSSSTSSTSSTHHHTSSSHHHSSSSSS
SSSHHSSSSSTSSTD

6m3m_A

NTASWFTALTQHGKEDLKFPRGQGVPINTNSSPDDQIGYYRRATRRIRXMKDLSPRWYFYYLGTGPEAGLPYGANKDGIIWVATEGALNTPKDHIGTRNPANNAAIVLQLP
QGTTLPKGFYAX

TSSSHSSSSSSTSHSSSSSSHHSTSSSSHHSSHHHTSSSSSSSSSSSTDTSSSSSSSSSSSSTSTHTHHSSSTSSSSTSSSSSSSTSSTSSTHHHTSSSHHHSSSSSSSSS
STSSSSSTSSTD

6yi3_A

GAMGLPNNTASWFTALTQHGKEDLKFPRGQGVPINTNSSPDDQIGYYRRATRRIRGGDGKMKDLSPRWYFYYLGTGPEAGLPYGANKDGIIWVATEGALNTPKDHIGTRNP
ANNAAIVLQLPQGTTLPKGFYAEGSRGGSX

TSSTSTSHTSSHSSSSSSSTHSHTSSSSTSTSSSTHHSSHHHHSSSSSSSSSSSSSHHTSSHTSSSSSSSSSSTSTHTHTSSSTSSSSTSSSSSSSTSSTSSTHHHHHSSH
HHSSSSSSSSSSSTSSSSSTSSSSTSSSTTD

8ctk_A

LEQWNLVIGFLFLTWICLLQFAYANRNRFLYIIKLIFLWLLWPVTLACFVLAAVYRINWITGGIAIAMACLVGLMWLSYFIASFRLFARTRSMWSFNPETNILLNVPLHGT
ILTRPLLESELVIGAVILRGHLRIAGHHLGRCDIKDLPKEITVATSRTLSYYKLGASQRVAGDSGFAAYSRYRIGNYX

THHHHHHHHHHHHHHHHHHHHHHHSSSHHHHHHHHHHHHHHHHHHHHHHHHHHHTSHSHHHHHHHHHHHHHHHHHHHHHHHHHHHHHHHTSHHHTSHHSTSSSSSTSTTS
SSSSSSHHTSHSSSSSSSTTSSSSTTSSHTSSSHHHSSHSSSSSSSTSSSSSSSSSSSSSTTHSSSSSSSSHSSSSTD

8ctk_B

LEQWNLVIGFLFLTWICLLQFAYANRNRFLYIIKLIFLWLLWPVTLACFVLAAVYRINWITGGIAIAMACLVGLMWLSYFIASFRLFARTRSMWSFNPETNILLNVPLHGT
ILTRPLLESELVIGAVILRGHLRIAGHHLGRCDIKDLPKEITVATSRTLSYYKLGASQRVAGDSGFAAYSRYRIGNYX

THHHHHHHHHHHHHHHHHHHHHHHSSSHHHHHHHHHHHHHHHHHHHHHHHHHHHTSHSHHHHHHHHHHHHHHHHHHHHHHHHHHHHHHHTSHHHTSHHSTSSSSSTSTTS
SSSSSSHHTSHSSSSSSSTTSSSSTTSSHTSSSHHHSSHSSSSSSSTSSSSSSSSSSSSSTTHSSSSSSSSHSSSSTD
